# Proteomics of thyroid tumours provides new insights into their molecular composition and changes associated with malignancy

**DOI:** 10.1038/srep23660

**Published:** 2016-03-30

**Authors:** Juan Martínez-Aguilar, Roderick Clifton-Bligh, Mark P. Molloy

**Affiliations:** 1 Department of Chemistry and Biomolecular Sciences, Macquarie University, NSW 2109, Australia; 2 Australian Proteome Analysis Facility, Macquarie University, NSW 2109, Australia; 3 Kolling Institute of Medical Research, Royal North Shore Hospital, St Leonards NSW 2065, Australia

## Abstract

Around 5% of the general population have palpable thyroid nodules. Although most thyroid tumours are benign, thyroid cancer represents the most common malignancy of the endocrine system, comprising mainly follicular and papillary thyroid carcinomas. Previous studies have shed some light on the molecular pathogenesis of thyroid cancer but there have not been any comprehensive mass spectrometry-based proteomic studies of large scale to reveal protein expression differences between thyroid tumours and the molecular alterations associated with tumour malignancy. We applied data-independent acquisition mass spectrometry which enabled quantitative expression analysis of over 1,600 proteins from 32 specimens to compare normal thyroid tissue with the three most common tumours of the thyroid gland: follicular adenoma, follicular carcinoma and papillary carcinoma. In follicular tumours, we found marked reduction of the tumour suppressor and therapeutic target extracellular protein decorin. We made the novel observation that TGFβ-induced protein ig-h3 (TGFBI) was found frequently overexpressed in follicular carcinoma compared with follicular adenoma. Proteomic pathway analysis showed changes in papillary carcinoma were associated with disruption of cell contacts (loss of E-cadherin), actin cytoskeleton dynamics and loss of differentiation markers, all hallmarks of an invasive phenotype.

Thyroid cancer is the most common endocrine malignancy. Around 5% of the general population have palpable thyroid nodules and this number increases to 50% with the use of ultrasonography^1^. Although most nodules are benign, there were estimated ~300,000 newly diagnosed thyroid cancer cases worldwide in 2012, 2.1% of all newly diagnosed cancers^2^. Most thyroid carcinomas arise from the thyroid follicular cells and the vast majority can be grouped into two histological types: follicular (10–15% of cases) and papillary (80–85% of cases) thyroid carcinoma or FTC and PTC, respectively, commonly referred to as differentiated thyroid carcinomas (DTC)^3^.

The World Health Organisation defines PTC as ‘a malignant epithelial tumour showing evidence of follicular cell differentiation and characterised by distinctive nuclear features’^4^. Accordingly, although the papillary architecture is a distinctive trait of classic PTC (two-thirds of cases), the formation of papillae is not a requirement for PTC diagnosis. On the other hand, the definition of FTC is based on malignancy and lack of the diagnostic nuclear features associated with PTC. About 50% of PTC cases present cervical lymph node metastasis (LNM) and distant spread is present in up to 20% of FTC. Both histotypes present good overall prognosis but survival rates drop in older patients and in those with distant metastasis^3^,^5^. The mainstay of treatment for patients diagnosed with thyroid cancer is thyroidectomy, followed by ablation of remnant tissue with ^131^I and thereafter thyroid hormone replacement therapy.

From a clinical standpoint, an ancillary tool is needed to improve the cytological evaluation of ultrasound-guided fine needle aspiration (FNA) biopsy of patients with thyroid nodules. Given that the identification of FTC is based on the capsular or vascular invasion of neoplastic cells, it is apparent that follicular adenoma (FA) and FTC are indistinguishable by FNA cytology. In fact, the latter is able to discriminate between benign and malignant tumours only in 70–80% of cases. Moreover, only 20–30% of thyroid nodules diagnosed as follicular neoplasms by FNA cytology (15–30% of biopsies) are found to be follicular carcinomas on histological examination, which means that most of those patients undergo unnecessary surgery^6^,^7^,^8^, with consequences of life-long hormone replacement. In light of the difficulties in the preoperative diagnosis of malignant thyroid neoplasms, it is clear that molecular markers of tumour malignancy would be very helpful.

Common genetic alterations in thyroid cancer are engaged in the mitogen-activated protein kinase (MAPK) and phosphatidylinositol 3-kinase (PI3K)/Akt signalling pathways. Classic PTC frequently presents *B-type Raf kinase* (*BRAF*) V600E mutation and rearranged in transformation/papillary thyroid carcinoma (*RET/PTC*) rearrangements whereas *RAS* mutations have been described in FA, FTC and the follicular variant of PTC. It is thought that FTC can develop from FA and that accumulation of genetic alterations leads to poorly differentiated and undifferentiated (anaplastic) carcinomas from DTC^3^,^5^.

The molecular background of PTC has been studied by immunohistochemistry and transcriptomic analyses while only limited proteomic studies have dealt with the analysis of specific thyroid cancer histotypes^5^,^9^,^10^,^11^,^12^,^13^. For example, Sofiadis *et al.* used two-dimensional gel electrophoresis of cytosolic tumour extracts to discover 25 proteins that discriminated follicular and papillary thyroid tumours^14^. In a separate study, this group also used SELDI-MS to show that S100A6 was often overexpressed in PTC compared with follicular thyroid tumours and normal tissue^15^. Newer proteomic approaches using advanced mass spectrometry enable deeper quantitative analyses revealing expression of thousands of proteins, sufficient to study biological pathways to improve our understanding in disease biology and propose new markers of diagnostic potential^16^,^17^,^18^.

In this work, we used a data-independent acquisition mass spectrometry method known as SWATH^17^,^18^,^19^ to perform a global proteomic quantitative comparison of FA, FTC, PTC and normal (N) thyroid tissue. This led to the identification of over 2,600 proteins, providing the most comprehensive molecular proteome description of thyroid tumours to date. From this data we could glean new biological pathway insights on the distinctive molecular changes in these three common tumours of the thyroid gland.

## Results

### Proteomics profiles of follicular and papillary thyroid tumours show marked differences in protein expression

**Figure 1:**
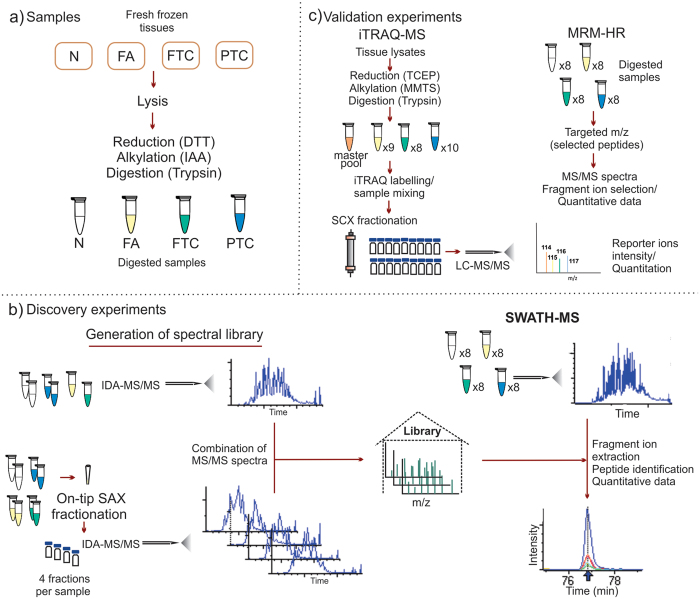
Overview of sample preparation and analysis. (**a**) Fresh frozen tissues from histologically normal (N), FA, FTC and PTC samples were lysed and subjected to protein reduction, alkylation and tryptic digestion; (**b**) generation of spectral library for SWATH-MS was achieved by combination of independent IDA-MS/MS experiments from six unfractionated and eight SAX-fractionated samples, which allowed downstream data processing including peptide identification and quantitative analysis; (**c**) iTRAQ-MS and MRM-HR were employed as validation techniques; the former was carried out by 4-plex labelling of FA, FTC, PTC and masterpool samples subsequently mixed, fractionated by SCX chromatography and loaded onto the LC-MS system. MRM-HR of selected proteins/peptides was performed targeting specific precursor m/z values, collecting high-resolution MS/MS spectra and extracting quantitative information from selected fragment ions.

**Figure 2:**
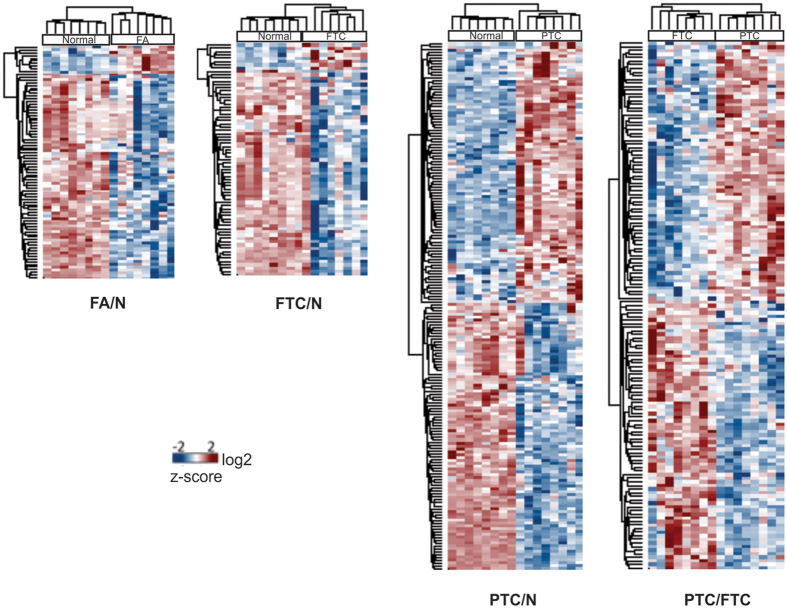
Hierarchical cluster analysis of differentially expressed proteins between thyroid tumours and histologically normal (N) thyroid tissue.

Figure 1 shows an overview of the sample preparation and mass spectrometry analysis carried out in this work. Using a subset of each thyroid tissue type we used mass spectrometry to establish a peptide spectral library of thyroid tissue that represented 2,682 proteins. Data-independent acquisition using SWATH mass spectrometry was then conducted for all 32 specimens (Supplementary Table S1). Quantitative values were able to be extracted for 1,629 proteins across all 32 samples, forming the basis for subsequent analyses. Hierarchical cluster analysis of protein expression based on ANOVA showed a clear separation of PTC tumours from all other sample groups and marked similarity between FA and FTC tumours (see Supplementary Fig. S1). Pairwise comparisons using histologically normal thyroid tissue as a reference group showed the number and pattern of molecular changes in FA and FTC to be clearly different from those in PTC tumours (see Fig. 2; original quantitative data can be found on the Supplementary dataset). Follicular tumours exhibited marked decreases in protein expression compared to normal thyroid tissue, whereas PTC samples showed different expression patterns of both over- and under-expression compared with normal tissue (Fig. 2). Out of a total of 86 and 71 differentially expressed proteins (fold-change fc ≥ 2) in FA and FTC, respectively, only 10 (FA) and 8 (FTC) were increased in these tumours (Supplementary Tables S2–S3). A rather different pattern was observed in PTC tumours which showed significant expression change of 184 proteins compared to normal thyroid tissue (91 up, 93 down) (Supplementary Table S4). Comparison of FA and FTC showed remarkable similarity of the respective proteomes, only TGFβ-induced protein ig-h3 (TGFBI) was elevated in FTC (fc = 3.6). Comparison of FTC with PTC tumours resulted in 147 differentially expressed proteins (Supplementary Table S5). These molecular protein profiles determined by SWATH-MS profiling are consistent with the distinct histological and clinicopathological characteristics of follicular and papillary tumours.

### Gene ontology and molecular pathway analyses

**Figure 3:**
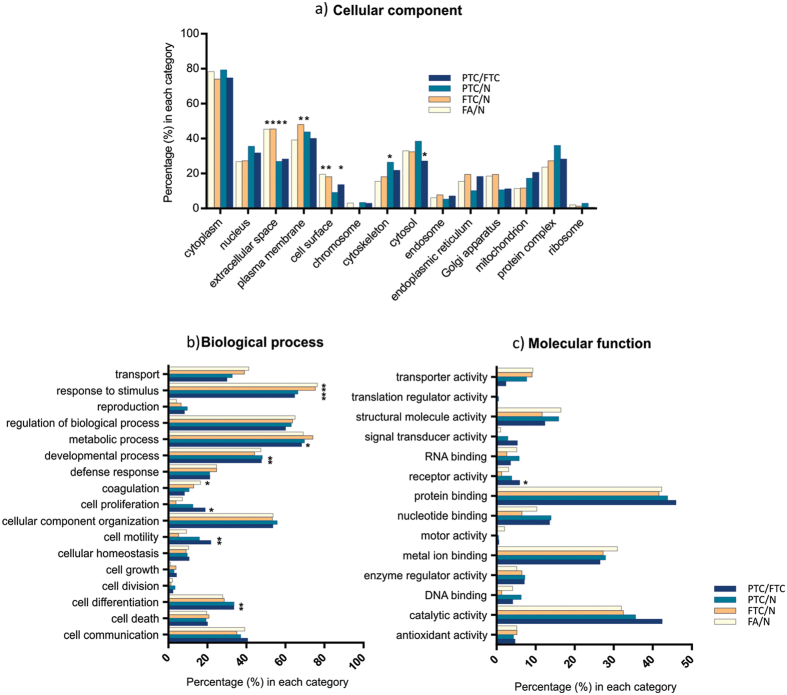
Gene Ontology analysis based on altered protein expression amongst sample groups. Results are presented for the three classifications: (**a**) cellular component, (**b**) biological process and (**c**) molecular function. Over-represented categories according to Fisher’s exact test (p < 0.01) are marked with*.

Gene Ontology enrichment analysis indicated over-representation, among others, of plasma membrane and extracellular proteins in the comparisons FTC/N and PTC/N (see Fig. 3). Additionally, the comparison PTC/N showed enrichment of cytoskeletal proteins and both PTC/N and PTC/FTC showed over-representation of the categories cell differentiation, cell motility and developmental process.

Canonical pathway analysis using the IPA software for the comparison FTC/N was led by the downregulation of acute phase and coagulation proteins including fibrinogen chains and alpha-2-macroglobulin (see Table 1). Although the expression of these proteins was also decreased in PTC with reference to normal samples, in the case of papillary tumours the top enriched pathways identified were ‘actin cytoskeleton signalling’ and ‘remodelling of epithelial adherens junctions’ (Table 1). Other enriched pathways were related to the activity of Rho family GTPases, which are known regulators of the actin cytoskeleton, cell-cell junctions and motility.

### Protein expression changes in follicular-patterned tumours

**Figure 4:**
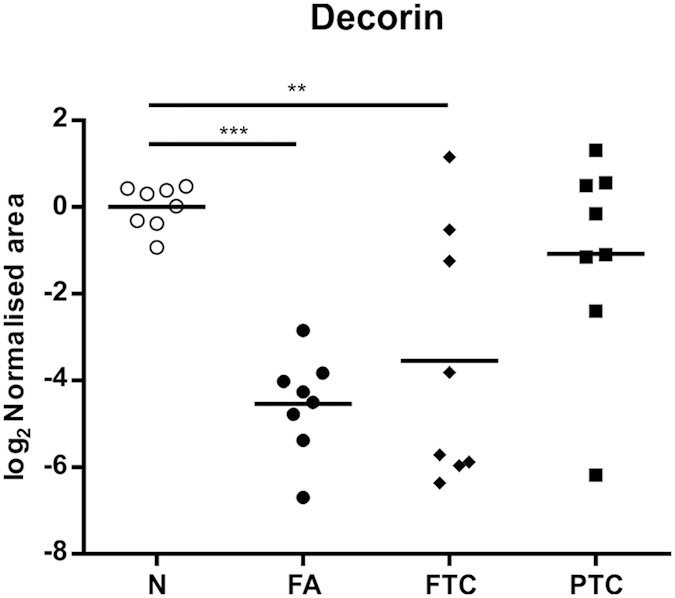
Relative expression of the extracellular protein decorin in FA, FTC and PTC tumours with reference to histologically normal (N) samples. ***p < 0.001, **p < 0.01.

**Figure 5:**
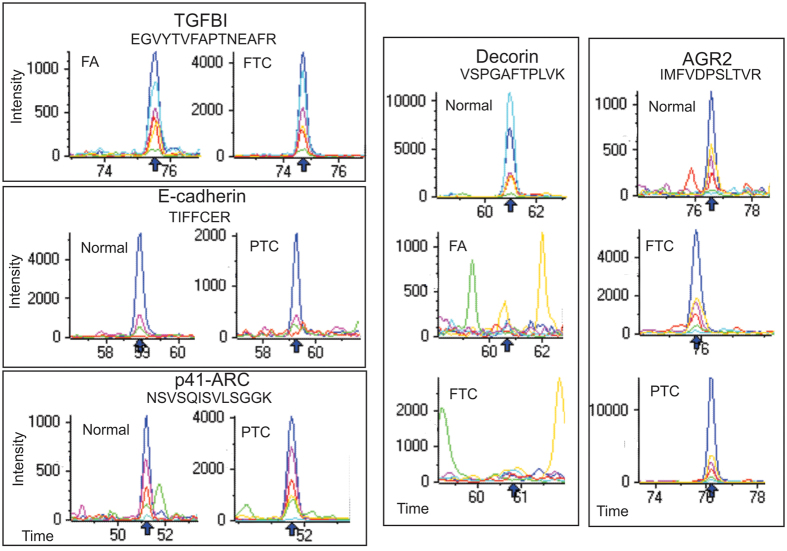
Extracted ion chromatograms (XIC) of example peptides (sequence shown) corresponding to different proteins in the sample groups. Arrow indicates peptide retention time (min) for ion extraction and intensity is ion counts per second. Coloured traces refer to XIC for various product ions for each peptide.

Both follicular tumour types showed marked reduction (>10-fold) of the extracellular tumour suppressor proteoglycan decorin or PGS2 (see Figs 4 and 5), negative regulator of receptor tyrosine kinases and growth factor signalling. Additionally, FTC showed altered expression of other extracellular proteins that have been associated with tumourigenesis such as collagen alpha-1 (XV) chain, serpin H1 and tenascin^20^,^21^,^22^.

**Figure 6:**
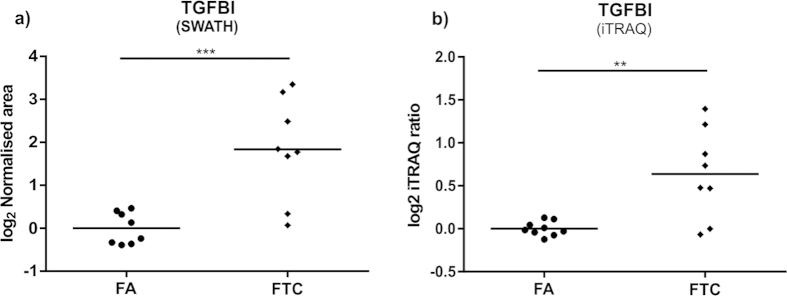
Relative expression of TGFBI in FA and FTC tumours as determined by (**a**) SWATH and (**b**) iTRAQ analysis; **p < 0.01, ***p < 0.001.

The comparison of FA with FTC tumours revealed that the 72 kDa extracellular protein TGFBI could correctly separate most FTC samples from the FA group with a relatively high fold change (see Fig. 6a). Subsequently, we used two alternative mass spectrometry approaches (isobaric stable-isotope labelling iTRAQ-MS, Fig. 6b) and high resolution multiple reaction monitoring (MRM-HR; Supplementary Fig. S2) to confirm the differential expression of TGFBI. The fact that the expression of most proteins remained unchanged between FA and FTC is a clear demonstration of the high similarity of the molecular phenotype of these two histotypes and underscores why it has been difficult to obtain a molecular signature of malignancy among tumours with follicular histology.

### PTC tumours exhibit several distinctive proteomic changes

The comparison of PTC tumours with normal samples readily identified many proteins commonly associated with the papillary histotype (e.g. annexin A1, thymosin β10, galectin-3, cytokeratin 19, ICAM-1, UDP-glucose 4-epimerase, cellular retinoic acid-binding protein 1, fibronectin, S100A6^5^,^9^,^10^,^11^,^12^,^15^,^23^,^24^; see Supplementary Table S4), hence validating the analyses shown here. Also, thyroid-specific proteins, thyroid peroxidase (TPO) and iodotyrosine dehalogenase 1 (DEHAL1) were found at reduced levels in PTC, a feature also previously observed by transcriptomic studies^9^. The expression of these proteins was not changed in follicular tumours. Many of the proteins with altered expression between PTC and normal samples were also identified in the comparison PTC/FTC (Supplementary Table S5).

### Altered expression of a common subgroup of proteins is found in malignant FTC and PTC tumours

**Figure 7:**
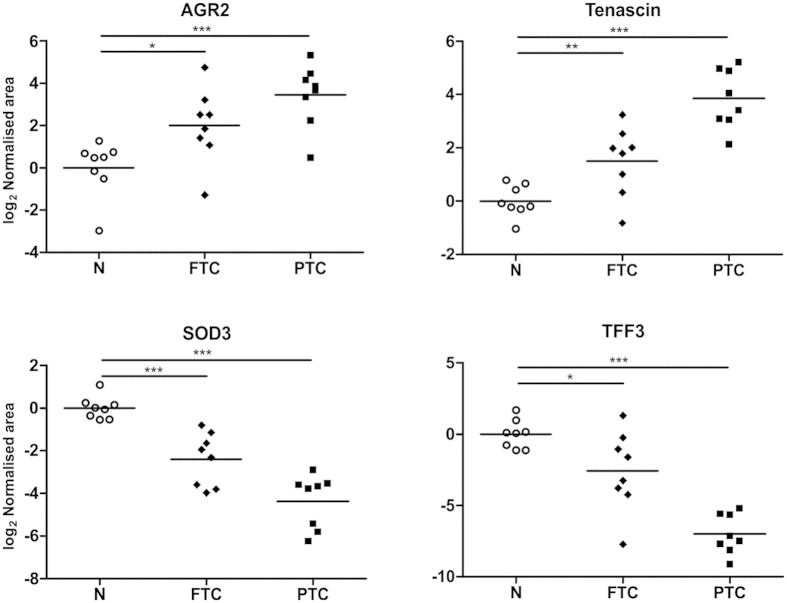
SWATH-MS relative expression of example proteins differentially expressed in both FTC and PTC tumours with reference to histologically normal (N) samples. ***p < 0.001, **p < 0.01, *p < 0.05.

FTC and PTC tumours shared dysregulation of 39 proteins with respect to normal thyroid tissue (Supplementary Table S6), including anterior gradient homolog 2 (AGR2), tenascin, extracellular superoxide dismutase (SOD3), 14-3-3 gamma, immunoglobulin superfamily containing leucine-rich repeat protein (ISLR), biotinidase and trefoil factor 3 (TFF3). Interestingly, the magnitude of expression of AGR2, tenascin, SOD3 and TFF3 was larger in PTC (see Fig. 7).

### Validation of protein expression

iTRAQ-MS analysis of FA, FTC and PTC samples confirmed the differential expression of most proteins (see Fig. 6 and Supplementary Table S7) and was well correlated with SWATH-MS (r^2^ = 0.84), serving as a general validation of the label-free quantitation approach. Additionally, the expression of several proteins (decorin, TGFBI, TPO, AGR2, SOD3, TFF3, tenascin, Arp2/3 proteins p41-ARC and p20-ARC, biotinidase, serpin H1 and E-cadherin) was further confirmed by targeted analysis using MRM-HR (see Supplementary Fig. S2).

**Figure 8:**
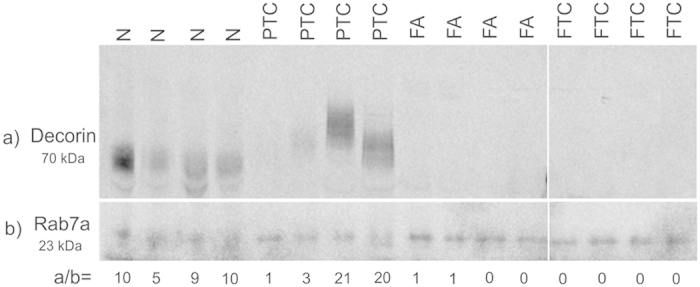
Western blot of decorin confirms decreased expression in FA and FTC samples when compared with histologically normal (N) and PTC samples. Rab7a protein was used as reference control^23^.

Western blot of decorin proteoglycan in normal, PTC, FA and FTC samples also confirmed loss of expression in tumours with follicular growth pattern FA and FTC (Fig. 8). The broad molecular weight bands observed in some patient samples are consistent with heterogeneous glycosylation^25^.

## Discussion

The significantly different number of protein changes and patterns of protein over- and under-expression in follicular and papillary tumours determined by mass spectrometry closely reflect known histological and clinicopathological features of thyroid tumours. Due to extensive quantitative comparison of over 1,600 proteins across 32 specimens our analysis provides the most comprehensive proteomic landscape analysis of thyroid tumours to date. Although FTC is more likely to metastasize to distant organs than PTC, this occurs with much less frequency than lymphatic metastasis in PTC. Since we studied only LNM-positive PTC cases, our results may help to explain the common metastatic phenotype of PTC as compared with the benign (FA) and minimally invasive follicular tumours (FTC).

A striking observation was the loss of the proteoglycan decorin in follicular tumours FA and FTC compared to non-neoplastic thyroid tissue. No significant change was seen in PTC, suggesting an important role of decorin in follicular thyroid neoplasia. Importantly, our novel protein expression data discovered by mass spectrometry and supported by immunoblot are consistent with a study that measured decorin mRNA abundance in thyroid tumours by quantitative polymerase chain reaction (qPCR). Arnaldi *et al.* found significant decrease of decorin mRNA in FA and FTC with respect to normal tissues but not in PTC^26^. Moreover, lower decorin mRNA levels were also found in the follicular variant of PTC, therefore associating decorin loss with follicular-patterned thyroid tumours. Decorin is the prototypic member of the small leucine-rich proteoglycans (SLRP) family and has been recognised as a multifaceted anti-oncogenic protein of the tumour microenvironment, regulating key cellular functions including proliferation, survival and migration^27^,^28^. Besides its participation in cellular matrix assembly by association with fibrillar collagens, it antagonistically targets multiple receptor tyrosine kinases (RTK), being therefore regarded as an endogenous pan-RTK inhibitor^28^. It also negatively regulates TGFβ signalling and has been found to induce paternally expressed gene 3 (Peg3), a master regulator of endothelial cell autophagy, thereby supporting decorin’s angiogenic suppressive activity^29^. Taken together, decorin loss facilitates tumour growth and progression and its markedly reduced expression in follicular thyroid tumours warrants further investigations. Several studies have shown that decorin reduces tumour growth and metastasis^30^,^31^,^32^,^33^ and is therefore currently considered an attractive therapeutic target that could also be useful in thyroid tumours of follicular histology.

Several molecular markers and mutational status have been proposed to aid in the diagnosis of thyroid tumour malignancy including a recent expression classifier employing 167 genes to distinguish benign thyroid nodules among those with indeterminate FNA cytology^34^. This suggests that several markers would be needed in order to detect follicular thyroid cancer. Indeed, we have found that the proteomic profiles of FA and FTC from our cohort are highly similar; however, we also found that the elevated expression of the extracellular protein TGFBI was able to differentiate 6 out of 8 FTC from FA specimens (Fig. 6), which comprise the two main types of thyroid tumours in the indeterminate category of FNA biopsies. Notably, increased TGFBI expression has also been shown in a mouse model of FTC progression. Kim *et al.* studied the changes in the gene expression profile of *Thrb*^*PV/PV*^ mice, which spontaneously develop metastatic FTC as they age. The TGFBI gene expression was dramatically upregulated during the course of the disease and up-regulation was confirmed at the protein level^35^.

Altogether, the consistent lower expression of TGFBI in all FA samples as compared to FTC cases, supported with previously observed *in vivo* upregulation, suggest that TGFBI is a protein that could be involved in the progression from thyroid adenoma to carcinoma and is therefore a novel candidate marker of malignancy in follicular thyroid tumours. Future investigations with larger sample cohorts will help to evaluate its diagnostic utility. It is interesting that TGFBI is known to participate in the dynamics of the extracellular space^27^,^28^, building upon the idea of a significant role of the tumour microenvironment in FTC, including loss of decorin.

As indicated by the pathway analysis, the comparison of follicular tumours with normal samples showed decreased expression of some acute phase proteins including members of the coagulation system. It is known that thyroid dysfunction influences the haemostatic balance and benign thyroid disorders such as hyperthyroidism and subclinical hypothyroidism have been linked to enhanced coagulation states^36^, which may explain the decreased expression of these proteins in these tumour samples. On the other hand, the comparison of PTC tumours with normal samples confirmed differential expression of several proteins commonly associated to this histotype. PTC tumours showed underexpression of thyroid function-related proteins TPO, DEHAL1 and thyroglobulin (TG), consistent with the de-differentiation of cellular function. Pathway analysis revealed differential expression of key proteins involved in cell migration and mapped to molecular pathways such as actin cytoskeleton signalling and remodelling of epithelial adherens junctions. Not unexpectedly, these changes were similarly observed in the comparison PTC/FTC, again highlighting the unique and divergent proteomic profile of papillary tumours.

Disruption of adherens junctions destabilises the cell-cell contacts and provides the cells with migratory potential, which in the case of tumour cells can lead to the onset of metastasis^37^. Consistent with their LNM-positive status, we have found that PTC tumours exhibit lower levels of E-cadherin, the core protein in adherens junctions. Our results and previous studies support the concept of E-cadherin as a tumour suppressor in PTC. Using immunofluorescence, Brabant *et al.* found frequent, albeit heterogeneous, reduction of E-cadherin in PTC and almost total loss in anaplastic thyroid carcinoma. LNM-positive samples showed undetectable immunoreactivity^38^. A separate study by Naito *et al.* employing immunohistochemistry (IHC) found decreased expression of E-cadherin in PTC compared with normal and FA tissues, an event more markedly seen in samples from patients with LNM^39^.

In PTC tumours, we also found overexpression of key proteins associated with remodelling of the actin cytoskeleton and cell locomotion. For example, several members of the Arp2/3 complex, actin, profilin-1, thymosin β4 and β10. The Arp2/3 complex is a master regulator of actin polymerisation at the leading edge of membrane protrusions of motile cells called lamellipodia^40^. Upregulated expression of Arp2/3 subunits has been found in both neoplastic and different stromal cells of invasive colorectal cancer^41^ and likewise the possible contribution of the stromal component in PTC should be acknowledged. Thymosin β4 and β10 are two polypeptides that act as actin sequesters that prevent polymerisation and form a reservoir of G-actin that can be released when needed to supply actin to filament ends^42^. Thymosin β10 expression has been correlated with tumour malignancy in thyroid cancer^43^. Other overexpressed proteins associated with the actin cytoskeleton and cell motility that we detected include coronin proteins and zyxin.

PTC and FTC tumours presented a group of common dysregulated proteins including SOD3, ISLR, biotinidase, polymerase I and transcript release factor (cavin-1), TFF3, alpha(B)-crystallin (CryAB), AGR2, tenascin and 14-3-3 gamma. Several of them showed larger expression change in PTC and it is tempting to speculate that it is related to the more aggressive LNM-positive PTC phenotype. Our results are in line with previous reports regarding the expression of several of these proteins in thyroid cancer. For example, an earlier study found decreased biotinidase expression in PTC tumours compared with benign tissues including follicular adenomas^44^. Nevertheless, we show here that biotinidase downregulation is common to both follicular and papillary tumours and is a potential general marker of thyroid neoplasia. Decreased expression of cavin-1 in FTC and PTC is in agreement with its downregulation in other tumours including prostate cancer where its expression has been shown to reduce cell migration, tumour growth, angiogenesis and lymphangiogenesis, therefore acting as a potential tumour suppressor^45^. Downregulation of carbonic anhydrases (CA) CA1, CA2 and CA4 was a common feature of thyroid tumours, which may be related to lower erythrocyte abundance since we found other erythrocyte proteins to be downregulated, including bisphosphoglycerate mutase; spectrin beta chain and haemoglobin subunits HBA, HBB and HBD.

Both thyroid carcinomas showed increased levels of other key proteins with known carcinogenic roles such as tenascin and AGR2. Tenascin elevation is in keeping with its high expression in most solid tumours and several reports suggesting involvement in tumour cell proliferation, angiogenesis and metastasis^22^. Other reports have documented the pro-oncogenic activity of AGR2 in various types of cancer, supported by *in vivo* experiments showing increased tumour growth and metastasis derived from AGR2-expressing cells^46^. Recent studies show that AGR2 acts as a thioredoxin in the endoplasmic reticulum (ER), interacting directly with EGFR for disulfide bond formation and protein folding and determining its presentation to the cell surface^47^. Functional EGFR overexpression has been found in thyroid cancer cells^48^ and AGR2 upregulation in PTC has also been demonstrated^49^.

In summary, we performed an extensive global proteomic comparison of the three most common thyroid tumours, PTC, FTC and FA. Importantly, besides our own confirmation of results by two mass spectrometry methods, several of our results are consistent with previous reports utilising immunohistochemical or mRNA expression analyses or *in vivo* functional studies. Extracellular matrix proteins were found to play a predominant role in the differential protein expression profiles of follicular thyroid tumours, particularly the loss of the anti-oncogenic protein and therapeutic target decorin. Moreover, we found that minimally invasive FTC tumours have frequent overexpression of TGFBI with respect to follicular adenomas, consistent with the results in a mouse model of FTC. PTC was characterised by destabilisation of cell-cell contacts, increased expression of key regulators of the actin cytoskeleton and under-expression of thyroid differentiation markers. Finally, through the use of comprehensive mass spectrometry-driven characterisation our study has provided a wealth of new information to further understand the molecular pathological basis of thyroid tumours.

## Methods

### Samples

Eight fresh frozen thyroid tissues from each sample group (normal histology, FA, minimally invasive FTC and classic PTC) were analysed by SWATH mass spectrometry. Two additional PTC samples and one additional FA sample were included for iTRAQ-MS analysis (Supplementary Table S1). All samples were obtained from the Kolling Institute of Medical Research, Sydney. Histologically normal samples were obtained from dissection of non-neoplastic tissue from the contralateral lobe of patients who had undergone thyroidectomy or from patients with benign disease where thyroidectomy was indicated. All PTC cases showed lymph node metastasis. The experimental protocols were approved by the human research ethics committees of the Northern Sydney Local Health District (1303–091M) and Macquarie University (5201300037). Informed consent was obtained from all subjects. All experiments were carried out in accordance with these approvals. Figure 1 shows an overview of the methods employed in this work.

### Sample preparation

All samples were lysed on ice with sonication in 20 mM HEPES, 1% sodium deoxycholate (SDOC), 0.1% SDS, 150 mM NaCl, and 1 mM EDTA, pH 8, with complete, EDTA-free protease inhibitor cocktail (Roche) and phosphatase inhibitor tablet (Thermo Scientific Pierce). Lysates were centrifuged (12,000 g, 10 min, 4 °C) and the supernatants retained. After protein quantitation (BCA method), 10 μg of protein were reduced in 10 mM dithiothreitol (DTT; 30 min, 60 °C), alkylated with 50 mM iodoacetamide (IAA; 30 min in the dark), diluted to 50 μL with 1% SDOC and incubated at 37 °C overnight with 0.2 μg of sequencing grade modified trypsin. After protein digestion, samples were acidified with 1 μL of formic acid and the precipitate was removed by centrifugation (14000 g, 10 min).

The spectral library for SWATH analysis was generated by combination of MS/MS spectra from six unfractionated (two normal, two PTC, one FA and one FTC) and eight strong anion-exchange (SAX) fractionated peptide samples (two from each thyroid tissue type). SAX fractionation was performed following the method described by Wiśniewski *et al.*^50^ at pH 11, 8, 5 and 3.

iTRAQ labelling was carried out using iTRAQ reagent 4-plex kit (AB Sciex). 100 μg of protein from all FA, FTC and PTC samples (including a masterpool of equal amounts of each sample) were treated with 5 mM tris-(2-carboxyethyl) phosphine (TCEP) for 1h at 60 °C and then with 10 mM cysteine blocking reagent, methyl methanethiosulfonate (MMTS) for 10 min at RT. Samples were diluted with 1% SDOC in 500 mM triethylammonium bicarbonate (TEAB) before trypsin digestion at 37 °C for 16 h. SDOC was removed by precipitation with formic acid and centrifugation (14000 g, 10 min) and the supernatants were dried in speed-vac and kept at −80 °C until use. Samples were analysed in groups consisting of a masterpool aliquot, a FA, a FTC and a PTC specimen. Peptide samples were reconstituted with 1M TEAB and labelled with iTRAQ isobaric tags in 100% ethanol for 1 h at RT. The reaction was quenched with Milli-Q water and labelled digests were combined in equal ratios. Before LC-MS analysis, desalted samples were fractionated by strong cation exchange with an Agilent 1260 LC, PolySULFOETHYL A column (200 × 2.1mm, 5um particle size) and UV detection at 210 nm. Weak solvent was 5 mM KH_2_PO_4_, pH 2.72, 25% v/v acetonitrile and strong solvent was 5 mM KH_2_PO_4_, pH 2.72, 350 mM KCl, 25% v/v acetonitrile. Samples were resuspended in weak solvent and fractionated with a gradient of strong solvent (0% for 25 min then 10–45% in 70 min and 45–100% in 5 min) at flow rate 0.3 mL/min. In total, 38 fractions of varying volume were collected and dried in a speed-vac.

### LC-MS analysis

10 μL of sample were loaded onto a ChromXP C18 trap (200 μm i.d. × 0.5 mm, 3 μm particle size) with an Ekspert nanoLC 400 cHiPLC system. Peptides were then separated on a ChromXP C18 column (200 μm i.d. × 15 cm, 3 μm particle size) with a 120 min gradient using 0.1% FA/2% ACN (v/v) in water and 0.1% FA (v/v) in ACN as weak and strong elution solvents, respectively. The LC system was coupled to a TripleTOF 6600 mass spectrometer (AB Sciex), where we employed SWATH-MS. To this end, we first built a reference spectral library by information-dependent analysis (IDA) of six different samples (two normal, two PTC, one FA and one FTC) and the eight samples prepared by SAX fractionation (two from each thyroid tissue type). Mass spectrometry conditions during IDA analysis were as follows: spray voltage-2.5 kV, interface heater temperature-150 °C, survey scan time-250 ms over the *m/z* range 350–1500, MS/MS with rolling collision energy of the 20 most intense precursor ions with charge states 2+ to 4+ and above 200 c.p.s., MS/MS accumulation time-100 ms in high sensitivity mode and dynamic exclusion of 30 s within 50 ppm. For SWATH-MS, 100 windows of variable *m/z* range and 50 ms accumulation time were employed, covering the precursor mass range of 350–1500 *m/z*. MS/MS scans with accumulation time of 30 ms were acquired in high sensitivity mode, resulting in a total cycle time of ~3.1 s and 8–10 points on average across peaks.

For the iTRAQ-labelled samples, 18 SCX fractions from each sample were selected based on the UV responses and preliminary tests on the number of non-redundant proteins identified in each SCX fraction. The latter were resuspended in 40 μL of 0.1% TFA, 2% ACN (v/v) in water, loaded sequentially onto a peptide trap and separated on an C18 Protecol column (100mm × 150 μm, 3 μm) with a 2-hour gradient using 0.1% FA/90% ACN (v/v) as strong solvent and an Eksigent LC system at a flow rate of 600 nL/min. The LC system was coupled to a TripleTOF 5600 mass spectrometer (AB Sciex). The spray voltage was set at 2.5 kV, with interface heater temperature of 150 °C. Mass spectrometry analysis was performed in IDA mode selecting the 10 most intense precursor ions over the m/z range 400–1500 with survey scan time of 250 ms at high resolution mode. MS/MS accumulation time was 200 ms, recorded in high sensitivity mode. Ions with 2+ to 5+ charge states were selected for MS/MS with dynamic exclusion of 20 s within 50 ppm, rolling collision energy and iTRAQ collision energy adjustment selected.

For the MRM-HR analyses, LC conditions were the same as in SWATH-MS. Peptide sequences and MS conditions can be found on Supplementary Table S8.

### Protein identification and quantitative analysis

IDA MS files from the groups of SAX-fractionated and unfractionated samples were processed using ProteinPilot 5.0 software with the Paragon database search algorithm. MS/MS spectra were searched against the UniProtKB/Swiss-Prot human protein database 2014_04 with 20,265 entries. Up to two missed cleavages were allowed and identification was performed considering cysteine alkylation by iodoacetamide, biological modifications and protein minimum confidence score of 95% (detected protein threshold >1.3) with FDR analysis enabled. The Paragon group files were imported into PeakView 2.1 software with SWATH acquisition MicroApp 2.0, shared peptides were excluded and the ion libraries from unfractionated and fractionated samples were exported to create an extended ion library using the list of ions from unfractionated samples as a seed spectral library. Only the following peptide modifications were kept: cysteine alkylation and protein N-terminal acetylation. A quality check was carried out to ensure that the relative intensities of fragment ions matched between the seed and add-on libraries. The precursor retention time of peptides passing these criteria was aligned to the seed library by supervised learning-based linear regression modelling on common peptides. For peptides with conflicting or overlapping spectra between seed and add-on libraries, only seed library peptides were kept and protein accessions were consolidated in an extended spectral library. The latter was then used for peptide identification in the SWATH files considering up to 100 peptides per protein, 6 transitions per peptide, 99% peptide confidence, 1% FDR, fragment ion extraction window of 10 min and mass tolerance of 75 ppm. In the SWATH acquisition MicroApp, selection of fragment ions depends on *m/z* and intensity, giving priority to ions of the greatest intensity above the precursor mass window and ions above y3 and b3 ions. The lists of identified proteins, peptides and their corresponding peak area and FDR were exported and the data were further filtered to keep only peptides with FDR ≤1% in at least five out of eight samples in each group (normal, FA, FTC, PTC) and remove reverse protein hits.

Data from the iTRAQ analysis were processed using the ProteinPilot 4.2 software using the following parameters: sample type – iTRAQ 4-plex; cysteine alkylation - MMTS; digestion – trypsin; ID focus - biological modifications and amino acid substitutions; search effort – thorough ID. FDR analysis was carried out with detected protein threshold (Unused ProtScore) set at 0.05. Automatic bias correction was selected for calculation of protein ratios. Proteins corresponding to local FDR <5% and global FDR <1% were selected for further analyses.

### Statistical analysis

The statistical analysis of SWATH data was performed on Perseus 1.5. Peak areas were log-transformed and normalised based on the median value in each of the thirty-two samples. Differentially expressed proteins in pairwise comparisons were identified by two-sided t-test (p < 0.05) and fold change fc ≥2. To account for multiple testing, we applied a permutation-based FDR analysis and report the corresponding q values. Spectral data of proteins with only one peptide were inspected manually and kept only the ones with clear peak co-elution, peak shape similarity and distinction from the noise level (see Supplementary Fig. S3 for some examples). To compare all four sample groups we applied one-way ANOVA p < 0.05. Heat maps were generated by hierarchical clustering with Euclidean distance and average linkage. For the iTRAQ validation data, protein abundances in each tumour type were first compared against those in the masterpool for each iTRAQ run and change of protein expression was identified based on a two-sided t-test p-value < 0.05 carried out on log-transformed iTRAQ ratios, considering only proteins detected in more than half of the cases and iTRAQ ratio >1.2.

### Gene ontology and pathway analysis

The list of proteins across all four sample groups and the differentially expressed sets of proteins from all comparisons were annotated and summarized at various gene ontology categories using the PloGO R package^51^. Each set of differentially expressed proteins was compared to the global set of identified proteins using the same software and enriched categories were identified based on a Fisher exact test p-value < 0.01. Protein placement in canonical pathways was undertaken using QIAGEN’s Ingenuity® Pathway Analysis (IPA®, QIAGEN Redwood City, www.qiagen.com/ingenuity).

### Western blot

Immunobloting was performed as previously described^23^. Briefly, 15 μg of protein were separated on 4–12% Bis-Tris NuPAGE gel and electroblotted onto nitrocellulose. The membrane was blocked with 5% skim milk, cut in two sections around 30 kDa and incubated overnight at 4 °C with either mouse monoclonal anti-decorin (1 μg/mL, clone 115402 R&D Systems) or rabbit monoclonal anti-Rab7a (1:500, Cell signalling). The secondary antibodies were fluorescence-labelled IRDye 800CW donkey anti-mouse and goat anti-rabbit IgG (LI-COR), incubated at RT for 1 h, visualised and quantitated using an Odyssey infrared imaging system and software(LI-COR, Lincoln, NE). Densitometry values were obtained using a fixed size rectangle feature enclosing the band of interest to obtain the intensity output after background subtraction (median intensity of pixels around the rectangle area). Measured values for decorin were divided by Rab7a values (reference control^23^) to compare decorin expression across samples.

## Additional Information

**How to cite this article**: Martínez-Aguilar, J. *et al.* Proteomics of thyroid tumours provides new insights into their molecular composition and changes associated with malignancy. *Sci. Rep.* **6**, 23660; doi: 10.1038/srep23660 (2016).

## Supplementary Material

Supplementary Information

Supplementary Dataset

## Figures and Tables

**Table 1:** Top five enriched canonical pathways in the proteomic comparison of FTC/N and PTC/N.

Name	p-value	Overlap	Molecules
FTC/N			
Acute phase response signaling	2.19E-07	4.8%	A2M,APCS,C4BPA,FGA,FGB,FGG,HP,RRAS
LXR/RXR Activation	6.24E-06	5.0%	APOB,APOC3,CLU,FGA,LYZ,S100A8
Coagulation system	8.81E-06	11.4%	A2M,FGA,FGB,FGG
Extrinsic prothrombin activation pathway	2.76E-05	18.8%	FGA,FGB,FGG
Atherosclerosis signaling	9.25E-05	4.1%	APOB,APOC3,CLU,LYZ,S100A8
PTC/N			
Actin Cytoskeleton Signaling	2.09E-11	8.1%	ACTB,ACTG1,ACTR2,ARPC1B,ARPC3,ARPC4,ARPC5,FN1,GNG12,GSN,MSN,MYH11,MYL4,PFN1,RAC2, TMSB10/TMSB4X,WASF2
Remodeling of epithelial adherens junction	7.00E-10	15.2%	ACTB,ACTG1,ACTR2,ARPC1B,ARPC3,ARPC4,ARPC5,CDH1,TUBA4A,ZYX
Regulation of actin-based motility by Rho	1.12E-08	11.5%	ACTB,ACTR2,ARPC1B,ARPC3,ARPC4,ARPC5,GSN,MYL4,PFN1,RAC2
RhoGDI signaling	1.20E-08	7.6%	ACTB,ACTG1,ACTR2,ARHGDIB, ARPC1B,ARPC3,ARPC4,ARPC5,CDH1,GNG12,MSN,MYL4,WASF2
Epithelial Adherens Junction Signaling	1.38E-08	8.4%	ACTB,ACTG1,ACTR2,ARPC1B,ARPC3,ARPC4,ARPC5,CDH1,MYH11,MYL4, TUBA4A,ZYX

The overlap represents the number of differentially expressed proteins divided by the total number of molecules in the canonical pathway.
