# Supplementary material for: Proteomics of thyroid tumours provides new insights into their molecular composition and changes associated with malignancy
Source: Sci Rep. 2016 Mar 30;6:23660. doi: 10.1038/srep23660 (PMC4812243; doi:10.1038/srep23660)
Supplement: Supplementary Information [file srep23660-s1.doc]

**Proteomics of thyroid tumours provides new insights into their molecular composition and changes associated with malignancy**

Juan Martínez-Aguilar^1,2^, Roderick Clifton-Bligh^3^ and Mark P. Molloy^1,2^*

^1^ Department of Chemistry and Biomolecular Sciences, Macquarie University, NSW 2109, Australia.

^2^ Australian Proteome Analysis Facility, Macquarie University, NSW 2109, Australia

^3^ Kolling Institute of Medical Research, Royal North Shore Hospital, St Leonards NSW 2065, Australia

**Supplementary Information**

**Supplementary Fig. S1.** Hierarchical clustering and heatmap from one-way anova (p<0.05) of SWATH-MS protein expression data in histologically normal (N) thyroid tissues, FA, FTC and PTC tumours


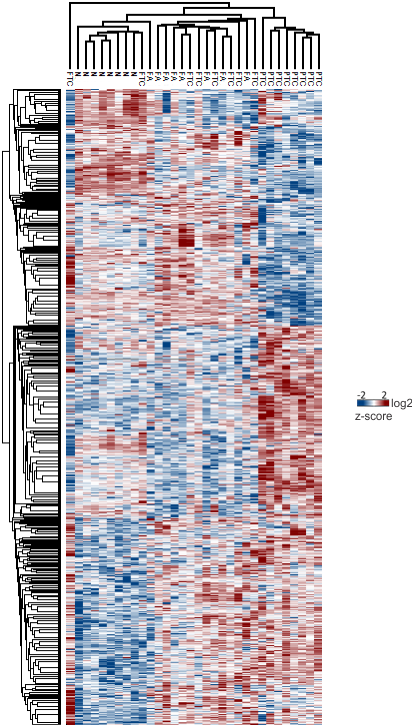


**Supplementary Fig. S2. Differential expression of proteins listed in Supplementary Table S8 in histologically normal thyroid and tumour tissues, analysed by MRM-HR.** Expression values were normalised against RAB7A and Cyclophilin A (PPIA_HUMAN) proteins following findings on Martinez-Aguilar, J., Clifton-Bligh, R. & Molloy, M.P. *BMC Cancer*. **15**, 199 (2015). Values were log-transformed and significant changes of protein expression were identified by either unpaired two-sided t-test or one-way anova followed by Holm-Sidak’s multiple comparison test with normal (N) samples as reference (*p < 0.05, **p < 0.01, ***p < 0.001)

**Supplementary Fig. S3.** Extracted ion chromatograms and matched MS/MS spectra of selected proteins with single-peptide quantification

Trefoil factor-3 (TFF3). EEYVGLSANQC[CAM]AVPAKDR (+3)

Extracted ion chromatogram (XIC)


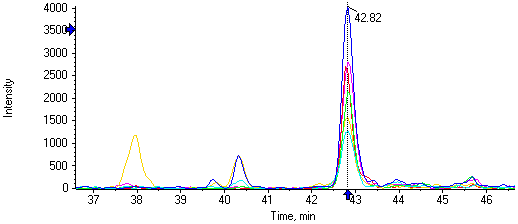


SWATH-MS spectrum (top) matching to IDA-MS based spectrum (bottom)


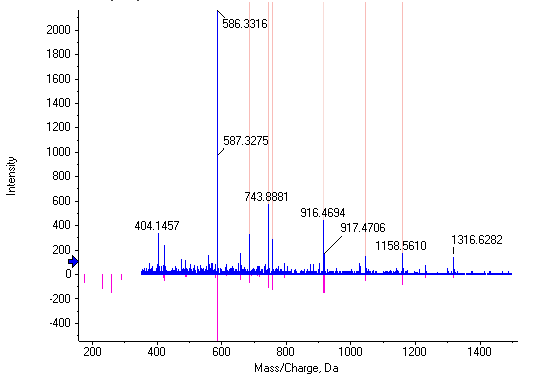


Biotinidase (BTD). LSSGLVTAALYGR (+2)

Extracted ion chromatogram (XIC)


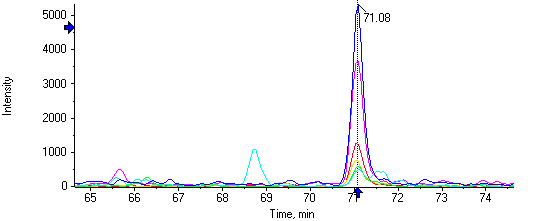


SWATH-MS spectrum (top) matching to IDA-MS based spectrum (bottom)


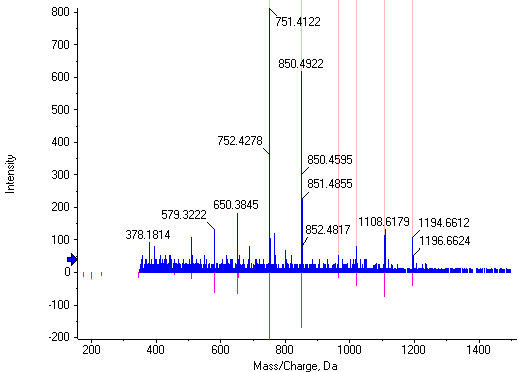


**UDP-glucose 4-epimerase** (GALE). AVGESVQKPLDYYR (+3)

Extracted ion chromatogram (XIC)


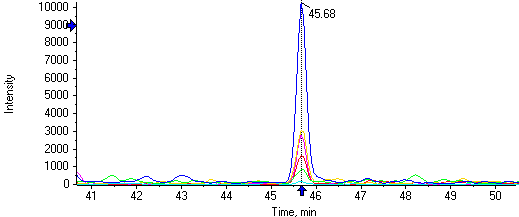


SWATH-MS spectrum (top) matching to IDA-MS based spectrum (bottom)


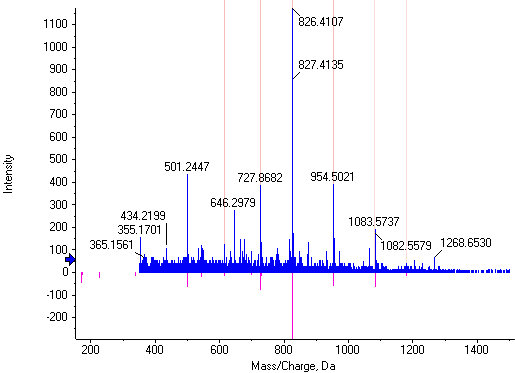


**Supplementary Table S1**. Clinicopathological features of the thyroid tissue samples analysed

| Normal tissues | | | | Follicular adenomas | | | |  |
| --- | --- | --- | --- | --- | --- | --- | --- | --- |
|  | Age | Sex |  |  | Age | Sex | Tumour  Size (mm) |  |
| Normal.1 | 42 | F |  | FA.1 | 36 | M | 30 |  |
| Normal.2 | 20 | F |  | FA.2 | 51 | F | 15 |  |
| Normal.3 | 78 | F |  | FA.3 | 30 | F | 30 |  |
| Normal.4 | 61 | M |  | FA.4 | 46 | F | 50 |  |
| Normal.5 | 51 | M |  | FA.5 | 14 | F | 8 |  |
| Normal.6 | 34 | F |  | FA.6 | 21 | M | 21 |  |
| Normal.7 | 84 | M |  | FA.7 | 68 | F | 8 |  |
| Normal.8 | 35 | M |  | FA.8 | 59 | F | 10 |  |
|  |  |  |  | FA.9* | 46 | M | 30 |  |
| Follicular thyroid carcinomas | | | | | | | |  |
|  | Age of onset | Sex | Tumour size (mm) | T stage | Capsular  invasion | Vascular  invasion |  |  |
| FTC.1 | 43 | F | 50 | 3 | no | yes |  |  |
| FTC.2 | 37 | M | 40 | 3 | yes | yes |  |  |
| FTC.3 | 82 | M | 45 | 3 | encapsulated | yes |  |  |
| FTC.4 | 16 | M | 13 | 1 | yes | no |  |  |
| FTC.5 | 64 | F | 40 | 2 | no | yes |  |  |
| FTC.6 | 64 | F | 25 |  | yes | no |  |  |
| FTC.7 | 20 | M | 110 | 3 | no | yes |  |  |
| FTC.8 | 22 | M | 30 | 2 | yes | yes |  |  |
| Papillary thyroid carcinomas | | | | | | | |  |
|  | Age of  onset | Sex | Tumour size  (mm) | T stage | Vascular  invasion | Extra-thyroidal  spread |  |  |
| PTC.1 | 24 | F | 30 | 2 | yes | no |  |  |
| PTC.2 | 50 | M | 6 | 4 | no | yes |  |  |
| PTC.3 | 59 | M | 30 | 3 | yes | yes |  |  |
| PTC.4 | 33 | F | 8 | 1 | no | no |  |  |
| PTC.5 | 24 | F | 45 | 3 | no | no |  |  |
| PTC.6 | 33 | F | 28 | 3 | yes | yes |  |  |
| PTC.7 | 49 | M | 44 | 3 | no | yes |  |  |
| PTC.8 | 56 | M | 55 | 4 | yes | yes |  |  |
| PTC.9* | 34 | F | 18 | 2 | no | no |  |  |
| PTC.10* | 32 | F | 55 | 4 | yes | yes |  |  |

* For iTRAQ-MS only

**Supplementary Table S2.** Proteins differentially expressed between FA/N as determined by SWATH. Shading indicates proteins increased in expression in FA.

| **Uniprot** | **Protein** | **log2(fc)** | **p-value** | **q-value** |
| --- | --- | --- | --- | --- |
| P07585 | PGS2_HUMAN | -4.4 | 8.3E-08 | 0.0013 |
| P08123 | CO1A2_HUMAN | -4.0 | 1.0E-04 | 0.0007 |
| P51888 | PRELP_HUMAN | -3.6 | 3.7E-05 | 0.0003 |
| P02743 | SAMP_HUMAN | -3.5 | 9.8E-06 | 0.0002 |
| P00325 | ADH1B_HUMAN | -3.4 | 4.1E-04 | 0.0019 |
| P69892 | HBG2_HUMAN | -3.2 | 1.8E-02 | 0.0360 |
| P20774 | MIME_HUMAN | -3.1 | 5.3E-06 | 0.0003 |
| P08294 | SODE_HUMAN | -2.9 | 1.4E-04 | 0.0012 |
| O14498 | ISLR_HUMAN | -2.9 | 1.4E-03 | 0.0044 |
| P68871 | HBB_HUMAN | -2.7 | 5.2E-03 | 0.0121 |
| P26447 | S10A4_HUMAN | -2.6 | 3.2E-04 | 0.0017 |
| P02042 | HBD_HUMAN | -2.6 | 5.5E-03 | 0.0130 |
| P61626 | LYSC_HUMAN | -2.5 | 1.2E-03 | 0.0038 |
| P22105 | TENX_HUMAN | -2.4 | 1.1E-08 | 0.0000 |
| P02730 | B3AT_HUMAN | -2.4 | 3.6E-03 | 0.0090 |
| Q86XX4 | FRAS1_HUMAN | -2.4 | 3.2E-05 | 0.0003 |
| P07355 | ANXA2_HUMAN | -2.4 | 2.6E-05 | 0.0003 |
| P02679 | FIBG_HUMAN | -2.3 | 4.4E-04 | 0.0018 |
| P69905 | HBA_HUMAN | -2.3 | 1.0E-02 | 0.0217 |
| Q16853 | AOC3_HUMAN | -2.3 | 2.8E-04 | 0.0016 |
| P01266 | THYG_HUMAN | -2.3 | 4.6E-02 | 0.0837 |
| Q9BXN1 | ASPN_HUMAN | -2.2 | 6.5E-03 | 0.0146 |
| P19971 | TYPH_HUMAN | -2.2 | 1.2E-04 | 0.0011 |
| Q86UN3 | R4RL2_HUMAN | -2.2 | 3.0E-03 | 0.0075 |
| P51884 | LUM_HUMAN | -2.2 | 1.1E-04 | 0.0007 |
| P00915 | CAH1_HUMAN | -2.2 | 7.2E-03 | 0.0158 |
| Q96CX2 | KCD12_HUMAN | -2.1 | 8.2E-06 | 0.0003 |
| P62805 | H4_HUMAN | -2.0 | 3.9E-03 | 0.0095 |
| P11166 | GTR1_HUMAN | -2.0 | 4.1E-03 | 0.0099 |
| P02671 | FIBA_HUMAN | -2.0 | 3.3E-04 | 0.0017 |
| P15291 | B4GT1_HUMAN | -2.0 | 1.1E-03 | 0.0034 |
| P02675 | FIBB_HUMAN | -1.9 | 1.1E-03 | 0.0033 |
| P04114 | APOB_HUMAN | -1.8 | 1.6E-03 | 0.0046 |
| P11277 | SPTB1_HUMAN | -1.8 | 7.8E-03 | 0.0171 |
| P04003 | C4BPA_HUMAN | -1.8 | 1.1E-03 | 0.0033 |
| P10909 | CLUS_HUMAN | -1.8 | 2.4E-03 | 0.0064 |
| Q5SZK8 | FREM2_HUMAN | -1.8 | 6.1E-03 | 0.0139 |
| Q01995 | TAGL_HUMAN | -1.7 | 2.6E-03 | 0.0066 |
| P00488 | F13A_HUMAN | -1.7 | 1.4E-03 | 0.0042 |
| P35749 | MYH11_HUMAN | -1.7 | 8.8E-04 | 0.0028 |
| P01023 | A2MG_HUMAN | -1.7 | 6.2E-03 | 0.0138 |
| P36269 | GGT5_HUMAN | -1.7 | 3.6E-04 | 0.0018 |
| Q09666 | AHNK_HUMAN | -1.6 | 4.4E-08 | 0.0020 |
| Q9NZN4 | EHD2_HUMAN | -1.6 | 5.9E-05 | 0.0006 |
| P01871 | IGHM_HUMAN | -1.6 | 7.2E-03 | 0.0159 |
| P02549 | SPTA1_HUMAN | -1.5 | 1.2E-02 | 0.0259 |
| Q07654 | TFF3_HUMAN | -1.5 | 5.9E-03 | 0.0135 |
| P00738 | HPT_HUMAN | -1.5 | 2.9E-03 | 0.0073 |
| Q02543 | RL18A_HUMAN | -1.5 | 4.9E-03 | 0.0112 |
| P01876 | IGHA1_HUMAN | -1.5 | 8.5E-03 | 0.0188 |
| P22748 | CAH4_HUMAN | -1.5 | 6.2E-03 | 0.0137 |
| P02751 | FINC_HUMAN | -1.5 | 2.7E-03 | 0.0068 |
| P22352 | GPX3_HUMAN | -1.5 | 7.2E-03 | 0.0157 |
| P00918 | CAH2_HUMAN | -1.4 | 1.8E-02 | 0.0367 |
| P15090 | FABP4_HUMAN | -1.4 | 3.8E-04 | 0.0019 |
| P59666 | DEF3_HUMAN | -1.4 | 2.3E-02 | 0.0454 |
| P32119 | PRDX2_HUMAN | -1.4 | 1.3E-03 | 0.0039 |
| P21810 | PGS1_HUMAN | -1.4 | 6.9E-03 | 0.0154 |
| P07305 | H10_HUMAN | -1.3 | 3.1E-04 | 0.0017 |
| P07738 | PMGE_HUMAN | -1.2 | 3.0E-02 | 0.0576 |
| P17931 | LEG3_HUMAN | -1.2 | 4.1E-03 | 0.0099 |
| Q6NZI2 | PTRF_HUMAN | -1.2 | 1.3E-06 | 0.0007 |
| P30043 | BLVRB_HUMAN | -1.2 | 4.5E-03 | 0.0107 |
| P43251 | BTD_HUMAN | -1.2 | 1.5E-03 | 0.0046 |
| P02656 | APOC3_HUMAN | -1.2 | 4.9E-02 | 0.0877 |
| P13987 | CD59_HUMAN | -1.1 | 4.7E-03 | 0.0111 |
| P13796 | PLSL_HUMAN | -1.1 | 5.9E-05 | 0.0006 |
| P06753 | TPM3_HUMAN | -1.1 | 2.4E-04 | 0.0016 |
| P09382 | LEG1_HUMAN | -1.1 | 2.9E-06 | 0.0005 |
| Q15582 | BGH3_HUMAN | -1.1 | 2.2E-05 | 0.0004 |
| P08603 | CFAH_HUMAN | -1.1 | 2.4E-03 | 0.0063 |
| O95865 | DDAH2_HUMAN | -1.0 | 1.1E-02 | 0.0246 |
| Q9UEY8 | ADDG_HUMAN | -1.0 | 1.1E-05 | 0.0004 |
| Q08380 | LG3BP_HUMAN | -1.0 | 2.2E-02 | 0.0436 |
| P51648 | AL3A2_HUMAN | -1.0 | 1.2E-02 | 0.0261 |
| O00159 | MYO1C_HUMAN | -1.0 | 5.7E-06 | 0.0003 |
| P09874 | PARP1_HUMAN | 1.0 | 5.3E-06 | 0.0003 |
| O43432 | IF4G3_HUMAN | 1.0 | 4.6E-02 | 0.0835 |
| Q9Y4L1 | HYOU1_HUMAN | 1.1 | 2.4E-03 | 0.0062 |
| P30101 | PDIA3_HUMAN | 1.1 | 2.9E-03 | 0.0073 |
| P11021 | GRP78_HUMAN | 1.1 | 1.5E-03 | 0.0045 |
| Q04837 | SSBP_HUMAN | 1.1 | 4.7E-03 | 0.0111 |
| P62306 | RUXF_HUMAN | 1.2 | 1.3E-02 | 0.0267 |
| P13667 | PDIA4_HUMAN | 1.2 | 1.8E-03 | 0.0050 |
| Q6PCB0 | VWA1_HUMAN | 1.3 | 1.5E-06 | 0.0006 |
| P14625 | ENPL_HUMAN | 1.3 | 8.4E-04 | 0.0029 |

**Supplementary Table S3. Proteins differentially expressed between FTC/N as determined by SWATH. Shading indicates proteins increased in expression in FTC.**

| **Uniprot** | **Protein** | **log2(fc)** | **p-value** | **q-value** |
| --- | --- | --- | --- | --- |
| P07585 | PGS2_HUMAN | -3.5 | 4.7E-03 | 0.0277 |
| O14498 | ISLR_HUMAN | -3.4 | 2.3E-04 | 0.0076 |
| P01266 | THYG_HUMAN | -3.2 | 1.0E-02 | 0.0416 |
| P68871 | HBB_HUMAN | -2.9 | 4.0E-03 | 0.0265 |
| P02743 | SAMP_HUMAN | -2.9 | 4.8E-04 | 0.0089 |
| P02042 | HBD_HUMAN | -2.9 | 2.3E-03 | 0.0200 |
| P22748 | CAH4_HUMAN | -2.8 | 3.0E-04 | 0.0093 |
| P69905 | HBA_HUMAN | -2.8 | 2.0E-03 | 0.0187 |
| Q8N5Z5 | KCD17_HUMAN | -2.8 | 1.5E-02 | 0.0521 |
| P00915 | CAH1_HUMAN | -2.6 | 5.8E-03 | 0.0305 |
| Q07654 | TFF3_HUMAN | -2.6 | 2.8E-02 | 0.0772 |
| P02730 | B3AT_HUMAN | -2.5 | 1.4E-03 | 0.0169 |
| P36269 | GGT5_HUMAN | -2.5 | 7.4E-04 | 0.0116 |
| P08294 | SODE_HUMAN | -2.4 | 1.8E-04 | 0.0087 |
| P11166 | GTR1_HUMAN | -2.1 | 4.3E-03 | 0.0258 |
| Q16853 | AOC3_HUMAN | -2.1 | 5.5E-03 | 0.0296 |
| P22105 | TENX_HUMAN | -2.1 | 2.5E-03 | 0.0208 |
| Q96CX2 | KCD12_HUMAN | -1.9 | 5.4E-03 | 0.0296 |
| P00325 | ADH1B_HUMAN | -1.9 | 3.1E-02 | 0.0845 |
| P11277 | SPTB1_HUMAN | -1.9 | 5.3E-03 | 0.0297 |
| P61626 | LYSC_HUMAN | -1.8 | 1.1E-02 | 0.0425 |
| P02679 | FIBG_HUMAN | -1.8 | 8.8E-03 | 0.0391 |
| P10909 | CLUS_HUMAN | -1.8 | 1.2E-03 | 0.0156 |
| P01871 | IGHM_HUMAN | -1.7 | 1.1E-02 | 0.0421 |
| P01023 | A2MG_HUMAN | -1.7 | 9.0E-04 | 0.0134 |
| P59666 | DEF3_HUMAN | -1.7 | 1.8E-02 | 0.0585 |
| P02549 | SPTA1_HUMAN | -1.6 | 4.3E-03 | 0.0261 |
| P02675 | FIBB_HUMAN | -1.6 | 1.5E-02 | 0.0518 |
| P17661 | DESM_HUMAN | -1.6 | 3.9E-02 | 0.0982 |
| P02671 | FIBA_HUMAN | -1.6 | 1.4E-02 | 0.0511 |
| Q86XX4 | FRAS1_HUMAN | -1.6 | 3.4E-02 | 0.0878 |
| Q9H3G5 | CPVL_HUMAN | -1.6 | 2.5E-02 | 0.0732 |
| P00918 | CAH2_HUMAN | -1.5 | 7.8E-03 | 0.0379 |
| Q86UN3 | R4RL2_HUMAN | -1.5 | 2.0E-02 | 0.0606 |
| Q9NZN4 | EHD2_HUMAN | -1.5 | 7.3E-03 | 0.0373 |
| P07355 | ANXA2_HUMAN | -1.4 | 1.8E-02 | 0.0586 |
| Q9H223 | EHD4_HUMAN | -1.4 | 1.2E-03 | 0.0157 |
| P04003 | C4BPA_HUMAN | -1.4 | 3.9E-03 | 0.0261 |
| Q12906 | ILF3_HUMAN | -1.4 | 4.7E-02 | 0.1126 |
| P07738 | PMGE_HUMAN | -1.4 | 1.2E-02 | 0.0451 |
| P02656 | APOC3_HUMAN | -1.3 | 2.8E-02 | 0.0773 |
| Q9BX97 | PLVAP_HUMAN | -1.3 | 1.1E-03 | 0.0149 |
| Q6NZI2 | PTRF_HUMAN | -1.3 | 7.4E-03 | 0.0367 |
| P30043 | BLVRB_HUMAN | -1.3 | 1.0E-03 | 0.0140 |
| P43251 | BTD_HUMAN | -1.3 | 4.4E-05 | 0.0020 |
| Q15847 | ADIRF_HUMAN | -1.3 | 3.9E-02 | 0.0982 |
| Q9Y646 | CBPQ_HUMAN | -1.3 | 2.7E-02 | 0.0753 |
| Q09666 | AHNK_HUMAN | -1.2 | 6.0E-03 | 0.0311 |
| P04114 | APOB_HUMAN | -1.2 | 1.3E-02 | 0.0472 |
| P01876 | IGHA1_HUMAN | -1.2 | 1.1E-02 | 0.0421 |
| P02511 | CRYAB_HUMAN | -1.2 | 1.9E-02 | 0.0599 |
| P39059 | COFA1_HUMAN | -1.2 | 4.6E-02 | 0.1098 |
| P05109 | S10A8_HUMAN | -1.2 | 2.5E-02 | 0.0732 |
| P32119 | PRDX2_HUMAN | -1.2 | 7.4E-03 | 0.0364 |
| P27105 | STOM_HUMAN | -1.2 | 1.8E-02 | 0.0582 |
| P00738 | HPT_HUMAN | -1.1 | 4.3E-02 | 0.1050 |
| P18135 | KV312_HUMAN | -1.1 | 1.2E-02 | 0.0436 |
| Q9H4G4 | GAPR1_HUMAN | -1.1 | 4.0E-02 | 0.0995 |
| P10301 | RRAS_HUMAN | -1.0 | 3.7E-02 | 0.0963 |
| Q13884 | SNTB1_HUMAN | -1.0 | 2.7E-02 | 0.0751 |
| P19971 | TYPH_HUMAN | -1.0 | 3.8E-02 | 0.0974 |
| P10768 | ESTD_HUMAN | -1.0 | 1.6E-03 | 0.0181 |
| P08729 | K2C7_HUMAN | -1.0 | 1.1E-02 | 0.0420 |
| Q9HDC9 | APMAP_HUMAN | 1.0 | 3.0E-03 | 0.0232 |
| P05787 | K2C8_HUMAN | 1.0 | 1.9E-02 | 0.0585 |
| P53992 | SC24C_HUMAN | 1.1 | 7.3E-03 | 0.0371 |
| P50454 | SERPH_HUMAN | 1.2 | 3.5E-04 | 0.0094 |
| Q08431 | MFGM_HUMAN | 1.2 | 2.1E-02 | 0.0635 |
| P61981 | 1433G_HUMAN | 1.4 | 2.6E-02 | 0.0757 |
| P24821 | TENA_HUMAN | 1.5 | 9.7E-03 | 0.0405 |
| O95994 | AGR2_HUMAN | 2.0 | 2.2E-02 | 0.0663 |

**Supplementary Table S4.** Proteins differentially expressed between PTC/N as determined by SWATH

| **Uniprot** | **Protein** | **log2(fc)** | **p-value** | **q-value** |
| --- | --- | --- | --- | --- |
| Q07654 | TFF3_HUMAN | -7.1 | 1.3E-08 | 0.0000 |
| Q8N5Z5 | KCD17_HUMAN | -6.6 | 5.0E-06 | 0.0001 |
| P01266 | THYG_HUMAN | -6.2 | 5.5E-07 | 0.0000 |
| P29762 | RABP1_HUMAN | -4.7 | 3.8E-05 | 0.0002 |
| P07202 | PERT_HUMAN | -4.5 | 6.5E-08 | 0.0000 |
| P08294 | SODE_HUMAN | -4.4 | 2.5E-07 | 0.0000 |
| P22748 | CAH4_HUMAN | -4 | 4.3E-08 | 0.0000 |
| P15090 | FABP4_HUMAN | -3.8 | 5.6E-05 | 0.0002 |
| O14498 | ISLR_HUMAN | -3.5 | 6.7E-06 | 0.0001 |
| Q86UN3 | R4RL2_HUMAN | -3.3 | 5.1E-07 | 0.0000 |
| P02743 | SAMP_HUMAN | -3.3 | 7.8E-05 | 0.0002 |
| Q6PHW0 | IYD1_HUMAN | -3.3 | 6.2E-05 | 0.0002 |
| O00339 | MATN2_HUMAN | -3.1 | 7.6E-08 | 0.0000 |
| P13473 | LAMP2_HUMAN | -3.1 | 2.5E-05 | 0.0001 |
| P12277 | KCRB_HUMAN | -3 | 6.1E-08 | 0.0000 |
| Q9Y646 | CBPQ_HUMAN | -2.9 | 3.4E-08 | 0.0000 |
| Q86XX4 | FRAS1_HUMAN | -2.9 | 1.4E-06 | 0.0000 |
| O94875 | SRBS2_HUMAN | -2.9 | 1.1E-08 | 0.0000 |
| Q5SZK8 | FREM2_HUMAN | -2.9 | 3.6E-08 | 0.0000 |
| P02511 | CRYAB_HUMAN | -2.9 | 1.1E-08 | 0.0000 |
| P21266 | GSTM3_HUMAN | -2.7 | 1.4E-06 | 0.0000 |
| P43251 | BTD_HUMAN | -2.7 | 1.4E-09 | 0.0000 |
| P69891 | HBG1_HUMAN | -2.6 | 9.1E-03 | 0.0085 |
| Q13642 | FHL1_HUMAN | -2.5 | 2.6E-08 | 0.0000 |
| P17661 | DESM_HUMAN | -2.3 | 2.0E-02 | 0.0200 |
| P15291 | B4GT1_HUMAN | -2.2 | 4.2E-04 | 0.0005 |
| P02042 | HBD_HUMAN | -2.2 | 2.7E-03 | 0.0025 |
| P22352 | GPX3_HUMAN | -2.2 | 2.2E-04 | 0.0003 |
| P00352 | AL1A1_HUMAN | -2.1 | 6.2E-07 | 0.0000 |
| Q13228 | SBP1_HUMAN | -2.1 | 2.8E-07 | 0.0000 |
| P69905 | HBA_HUMAN | -2.1 | 4.0E-03 | 0.0037 |
| P02730 | B3AT_HUMAN | -2 | 2.7E-03 | 0.0025 |
| Q13884 | SNTB1_HUMAN | -2 | 1.6E-03 | 0.0015 |
| Q9BX97 | PLVAP_HUMAN | -2 | 1.6E-07 | 0.0000 |
| P68871 | HBB_HUMAN | -2 | 4.3E-03 | 0.0039 |
| P50895 | BCAM_HUMAN | -2 | 2.4E-08 | 0.0000 |
| Q00796 | DHSO_HUMAN | -2 | 1.5E-04 | 0.0003 |
| P11277 | SPTB1_HUMAN | -1.9 | 9.8E-03 | 0.0091 |
| Q16853 | AOC3_HUMAN | -1.8 | 2.6E-03 | 0.0024 |
| Q9BR76 | COR1B_HUMAN | -1.8 | 2.6E-05 | 0.0001 |
| O15230 | LAMA5_HUMAN | -1.8 | 5.0E-05 | 0.0002 |
| P07942 | LAMB1_HUMAN | -1.8 | 1.7E-05 | 0.0000 |
| Q9GZM7 | TINAL_HUMAN | -1.7 | 9.2E-05 | 0.0002 |
| P23434 | GCSH_HUMAN | -1.7 | 2.4E-02 | 0.0243 |
| Q6UXI9 | NPNT_HUMAN | -1.7 | 1.9E-03 | 0.0018 |
| P08572 | CO4A2_HUMAN | -1.7 | 4.3E-05 | 0.0002 |
| O00584 | RNT2_HUMAN | -1.6 | 7.1E-05 | 0.0002 |
| Q9H223 | EHD4_HUMAN | -1.6 | 9.8E-09 | 0.0000 |
| P11166 | GTR1_HUMAN | -1.5 | 1.6E-03 | 0.0015 |
| P00915 | CAH1_HUMAN | -1.5 | 3.3E-02 | 0.0323 |
| P12830 | CADH1_HUMAN | -1.5 | 8.5E-06 | 0.0000 |
| P05026 | AT1B1_HUMAN | -1.5 | 1.1E-05 | 0.0000 |
| P30086 | PEBP1_HUMAN | -1.4 | 3.1E-05 | 0.0001 |
| Q08380 | LG3BP_HUMAN | -1.4 | 6.2E-05 | 0.0002 |
| Q6NZI2 | PTRF_HUMAN | -1.4 | 1.2E-06 | 0.0000 |
| P12109 | CO6A1_HUMAN | -1.4 | 1.6E-02 | 0.0160 |
| Q13425 | SNTB2_HUMAN | -1.4 | 6.9E-05 | 0.0002 |
| P00918 | CAH2_HUMAN | -1.3 | 3.0E-02 | 0.0299 |
| P30043 | BLVRB_HUMAN | -1.3 | 6.1E-04 | 0.0007 |
| O14495 | LPP3_HUMAN | -1.3 | 1.3E-06 | 0.0000 |
| P10768 | ESTD_HUMAN | -1.3 | 6.0E-07 | 0.0000 |
| Q96CM8 | ACSF2_HUMAN | -1.3 | 2.5E-03 | 0.0023 |
| P02679 | FIBG_HUMAN | -1.3 | 3.4E-02 | 0.0330 |
| P52597 | HNRPF_HUMAN | -1.3 | 1.4E-03 | 0.0014 |
| P05091 | ALDH2_HUMAN | -1.3 | 5.1E-06 | 0.0001 |
| Q9NZN4 | EHD2_HUMAN | -1.3 | 2.7E-04 | 0.0003 |
| P07738 | PMGE_HUMAN | -1.3 | 4.3E-02 | 0.0424 |
| P32119 | PRDX2_HUMAN | -1.2 | 4.5E-03 | 0.0042 |
| P01023 | A2MG_HUMAN | -1.2 | 1.1E-02 | 0.0104 |
| P27797 | CALR_HUMAN | -1.2 | 3.1E-06 | 0.0000 |
| Q04760 | LGUL_HUMAN | -1.2 | 1.7E-04 | 0.0003 |
| P17174 | AATC_HUMAN | -1.2 | 1.1E-04 | 0.0003 |
| P07305 | H10_HUMAN | -1.2 | 4.4E-05 | 0.0002 |
| Q9H0W9 | CK054_HUMAN | -1.2 | 9.2E-06 | 0.0000 |
| P10253 | LYAG_HUMAN | -1.1 | 3.0E-05 | 0.0001 |
| Q13510 | ASAH1_HUMAN | -1.1 | 2.5E-03 | 0.0023 |
| Q9UBI6 | GBG12_HUMAN | -1.1 | 1.7E-05 | 0.0000 |
| P35555 | FBN1_HUMAN | -1.1 | 2.5E-02 | 0.0249 |
| Q9HB40 | RISC_HUMAN | -1.1 | 2.3E-03 | 0.0021 |
| P14543 | NID1_HUMAN | -1.1 | 5.5E-06 | 0.0001 |
| P02748 | CO9_HUMAN | -1.1 | 2.6E-02 | 0.0254 |
| P49419 | AL7A1_HUMAN | -1.1 | 2.2E-04 | 0.0003 |
| P55268 | LAMB2_HUMAN | -1.1 | 1.8E-03 | 0.0017 |
| P36269 | GGT5_HUMAN | -1 | 6.5E-03 | 0.0059 |
| P11047 | LAMC1_HUMAN | -1 | 6.0E-05 | 0.0002 |
| P22105 | TENX_HUMAN | -1 | 1.1E-02 | 0.0105 |
| P00441 | SODC_HUMAN | -1 | 1.4E-05 | 0.0000 |
| Q14624 | ITIH4_HUMAN | -1 | 1.1E-02 | 0.0108 |
| Q9H8H3 | MET7A_HUMAN | -1 | 2.4E-03 | 0.0021 |
| Q9Y4L1 | HYOU1_HUMAN | -1 | 2.9E-03 | 0.0028 |
| P61204 | ARF3_HUMAN | -1 | 5.0E-03 | 0.0046 |
| P35749 | MYH11_HUMAN | -1 | 2.6E-02 | 0.0256 |
| P13716 | HEM2_HUMAN | -1 | 5.6E-03 | 0.0050 |
| O00299 | CLIC1_HUMAN | 1 | 1.9E-05 | 0.0000 |
| P13797 | PLST_HUMAN | 1 | 4.0E-03 | 0.0037 |
| P38606 | VATA_HUMAN | 1 | 4.3E-06 | 0.0001 |
| Q15436 | SC23A_HUMAN | 1 | 1.1E-06 | 0.0000 |
| P63261 | ACTG_HUMAN | 1 | 4.3E-06 | 0.0001 |
| P16401 | H15_HUMAN | 1 | 1.2E-02 | 0.0111 |
| P20700 | LMNB1_HUMAN | 1 | 1.8E-04 | 0.0003 |
| P62191 | PRS4_HUMAN | 1 | 6.4E-03 | 0.0058 |
| P54819 | KAD2_HUMAN | 1 | 5.6E-03 | 0.0050 |
| Q9Y6H1 | CHCH2_HUMAN | 1 | 7.4E-03 | 0.0069 |
| P04433 | KV309_HUMAN | 1 | 1.2E-02 | 0.0112 |
| P30050 | RL12_HUMAN | 1 | 6.4E-06 | 0.0001 |
| Q9Y6C9 | MTCH2_HUMAN | 1 | 2.3E-02 | 0.0234 |
| P06396 | GELS_HUMAN | 1 | 1.7E-08 | 0.0000 |
| P05386 | RLA1_HUMAN | 1 | 7.8E-05 | 0.0002 |
| P50552 | VASP_HUMAN | 1 | 2.7E-02 | 0.0269 |
| P12268 | IMDH2_HUMAN | 1.1 | 1.2E-06 | 0.0000 |
| Q9ULV4 | COR1C_HUMAN | 1.1 | 1.9E-04 | 0.0003 |
| P26038 | MOES_HUMAN | 1.1 | 3.0E-07 | 0.0000 |
| O15143 | ARC1B_HUMAN | 1.1 | 1.3E-06 | 0.0000 |
| Q15121 | PEA15_HUMAN | 1.1 | 9.1E-05 | 0.0002 |
| Q06323 | PSME1_HUMAN | 1.1 | 4.1E-06 | 0.0001 |
| P43304 | GPDM_HUMAN | 1.1 | 1.1E-02 | 0.0108 |
| O15145 | ARPC3_HUMAN | 1.1 | 6.1E-06 | 0.0001 |
| Q15942 | ZYX_HUMAN | 1.1 | 8.5E-03 | 0.0080 |
| O60437 | PEPL_HUMAN | 1.1 | 1.4E-03 | 0.0014 |
| Q9BRA2 | TXD17_HUMAN | 1.1 | 8.9E-08 | 0.0000 |
| P07737 | PROF1_HUMAN | 1.1 | 2.6E-06 | 0.0000 |
| P62263 | RS14_HUMAN | 1.1 | 1.5E-04 | 0.0003 |
| Q15165 | PON2_HUMAN | 1.1 | 5.7E-04 | 0.0007 |
| Q14764 | MVP_HUMAN | 1.1 | 4.2E-03 | 0.0039 |
| Q9Y6N5 | SQRD_HUMAN | 1.1 | 6.5E-03 | 0.0059 |
| P61160 | ARP2_HUMAN | 1.2 | 2.0E-06 | 0.0000 |
| Q9UJU6 | DBNL_HUMAN | 1.2 | 5.3E-05 | 0.0002 |
| P54727 | RD23B_HUMAN | 1.2 | 1.2E-04 | 0.0003 |
| P62136 | PP1A_HUMAN | 1.2 | 5.6E-04 | 0.0007 |
| P12829 | MYL4_HUMAN | 1.2 | 1.2E-02 | 0.0114 |
| P60709 | ACTB_HUMAN | 1.2 | 9.2E-05 | 0.0002 |
| O15511 | ARPC5_HUMAN | 1.2 | 1.1E-04 | 0.0003 |
| P25774 | CATS_HUMAN | 1.2 | 3.9E-02 | 0.0389 |
| P42765 | THIM_HUMAN | 1.2 | 1.1E-03 | 0.0011 |
| Q9UNM6 | PSD13_HUMAN | 1.2 | 1.4E-02 | 0.0130 |
| Q13596 | SNX1_HUMAN | 1.2 | 5.2E-05 | 0.0002 |
| Q9BWM7 | SFXN3_HUMAN | 1.3 | 3.1E-04 | 0.0003 |
| Q04837 | SSBP_HUMAN | 1.3 | 4.0E-04 | 0.0004 |
| P10412 | H14_HUMAN | 1.3 | 4.5E-02 | 0.0444 |
| Q8IV08 | PLD3_HUMAN | 1.3 | 7.2E-04 | 0.0008 |
| Q9Y3Z3 | SAMH1_HUMAN | 1.3 | 1.5E-03 | 0.0015 |
| Q86UX7 | URP2_HUMAN | 1.3 | 2.4E-02 | 0.0240 |
| Q07812 | BAX_HUMAN | 1.3 | 6.0E-04 | 0.0007 |
| Q9UHD8 | SEPT9_HUMAN | 1.3 | 1.3E-05 | 0.0000 |
| Q92598 | HS105_HUMAN | 1.4 | 5.3E-05 | 0.0002 |
| Q9Y6W5 | WASF2_HUMAN | 1.4 | 3.9E-02 | 0.0392 |
| P06703 | S10A6_HUMAN | 1.4 | 8.1E-03 | 0.0077 |
| P50454 | SERPH_HUMAN | 1.4 | 2.0E-06 | 0.0000 |
| P59998 | ARPC4_HUMAN | 1.4 | 4.6E-09 | 0.0000 |
| P52566 | GDIR2_HUMAN | 1.4 | 1.1E-03 | 0.0011 |
| Q14195 | DPYL3_HUMAN | 1.4 | 6.6E-05 | 0.0002 |
| O75223 | GGCT_HUMAN | 1.4 | 5.1E-03 | 0.0046 |
| Q15063 | POSTN_HUMAN | 1.4 | 4.2E-02 | 0.0418 |
| P13611 | CSPG2_HUMAN | 1.4 | 1.9E-02 | 0.0184 |
| Q96C19 | EFHD2_HUMAN | 1.4 | 6.9E-06 | 0.0001 |
| P42224 | STAT1_HUMAN | 1.5 | 7.2E-03 | 0.0067 |
| P68366 | TBA4A_HUMAN | 1.5 | 2.6E-06 | 0.0000 |
| P31949 | S10AB_HUMAN | 1.6 | 1.3E-05 | 0.0000 |
| P49961 | ENTP1_HUMAN | 1.6 | 2.5E-06 | 0.0000 |
| P61981 | 1433G_HUMAN | 1.6 | 1.0E-04 | 0.0003 |
| P80723 | BASP1_HUMAN | 1.7 | 7.5E-06 | 0.0001 |
| Q9UBG0 | MRC2_HUMAN | 1.7 | 4.2E-03 | 0.0039 |
| P17931 | LEG3_HUMAN | 1.7 | 2.9E-06 | 0.0000 |
| P04233 | HG2A_HUMAN | 1.8 | 2.7E-06 | 0.0000 |
| P15153 | RAC2_HUMAN | 1.8 | 7.6E-03 | 0.0073 |
| P05362 | ICAM1_HUMAN | 1.8 | 6.1E-04 | 0.0007 |
| P01857 | IGHG1_HUMAN | 1.8 | 9.7E-04 | 0.0010 |
| P0CG05 | LAC2_HUMAN | 1.8 | 2.7E-02 | 0.0272 |
| O95571 | ETHE1_HUMAN | 1.8 | 2.8E-04 | 0.0003 |
| P43243 | MATR3_HUMAN | 1.9 | 4.3E-03 | 0.0040 |
| Q95IE3 | 2B1C_HUMAN | 2 | 7.8E-05 | 0.0002 |
| P13796 | PLSL_HUMAN | 2 | 6.4E-05 | 0.0002 |
| P63313 | TYB10_HUMAN | 2.1 | 1.2E-09 | 0.0000 |
| P62328 | TYB4_HUMAN | 2.2 | 2.4E-07 | 0.0000 |
| P04083 | ANXA1_HUMAN | 2.5 | 4.3E-08 | 0.0000 |
| P24821 | TENA_HUMAN | 2.5 | 5.6E-05 | 0.0002 |
| P09758 | TACD2_HUMAN | 2.6 | 7.4E-03 | 0.0070 |
| Q14376 | GALE_HUMAN | 2.6 | 1.8E-05 | 0.0000 |
| P08727 | K1C19_HUMAN | 2.7 | 3.8E-06 | 0.0000 |
| P31146 | COR1A_HUMAN | 2.9 | 4.9E-04 | 0.0005 |
| Q8WU39 | MZB1_HUMAN | 2.9 | 3.4E-03 | 0.0031 |
| P02751 | FINC_HUMAN | 3.1 | 2.3E-04 | 0.0003 |
| O95994 | AGR2_HUMAN | 3.4 | 3.0E-04 | 0.0004 |
| Q8N6C5 | IGSF1_HUMAN | 3.4 | 1.4E-03 | 0.0013 |

**Supplementary Table S5.** Proteins differentially expressed between PTC/FTC as determined by SWATH

| **Uniprot** | **Protein** | **log2(fc)** | **p-value** | **q-value** |
| --- | --- | --- | --- | --- |
| Q07654 | TFF3_HUMAN | -4.5 | 1.2E-03 | 0.0076 |
| P07202 | PERT_HUMAN | -4.1 | 3.9E-05 | 0.0010 |
| Q8N5Z5 | KCD17_HUMAN | -3.9 | 9.6E-03 | 0.0315 |
| P12277 | KCRB_HUMAN | -3.5 | 7.1E-05 | 0.0008 |
| P01266 | THYG_HUMAN | -3.2 | 2.1E-02 | 0.0620 |
| P13473 | LAMP2_HUMAN | -2.9 | 1.2E-04 | 0.0015 |
| P21266 | GSTM3_HUMAN | -2.6 | 5.7E-07 | 0.0000 |
| P22352 | GPX3_HUMAN | -2.4 | 6.4E-03 | 0.0234 |
| Q86UN3 | R4RL2_HUMAN | -2.4 | 2.6E-03 | 0.0129 |
| Q00796 | DHSO_HUMAN | -2.4 | 9.9E-04 | 0.0068 |
| P15090 | FABP4_HUMAN | -2.3 | 4.7E-02 | 0.1211 |
| O00339 | MATN2_HUMAN | -2.2 | 1.4E-03 | 0.0084 |
| Q5SZK8 | FREM2_HUMAN | -2.2 | 9.8E-03 | 0.0319 |
| O94875 | SRBS2_HUMAN | -2.2 | 9.2E-06 | 0.0010 |
| Q6PHW0 | IYD1_HUMAN | -2.0 | 2.2E-02 | 0.0646 |
| P08294 | SODE_HUMAN | -2.0 | 5.4E-03 | 0.0222 |
| Q13642 | FHL1_HUMAN | -2.0 | 4.6E-05 | 0.0011 |
| Q9BT78 | CSN4_HUMAN | -2.0 | 1.9E-02 | 0.0581 |
| P50895 | BCAM_HUMAN | -1.9 | 1.5E-05 | 0.0008 |
| Q6UXI9 | NPNT_HUMAN | -1.9 | 1.3E-02 | 0.0425 |
| Q16363 | LAMA4_HUMAN | -1.8 | 3.8E-03 | 0.0169 |
| P02511 | CRYAB_HUMAN | -1.7 | 2.5E-03 | 0.0128 |
| Q96CM8 | ACSF2_HUMAN | -1.7 | 2.3E-03 | 0.0121 |
| P23434 | GCSH_HUMAN | -1.7 | 3.5E-02 | 0.0907 |
| Q9Y646 | CBPQ_HUMAN | -1.7 | 7.2E-03 | 0.0255 |
| Q13228 | SBP1_HUMAN | -1.7 | 2.9E-04 | 0.0032 |
| Q14112 | NID2_HUMAN | -1.6 | 5.1E-03 | 0.0211 |
| P30038 | AL4A1_HUMAN | -1.6 | 1.3E-02 | 0.0420 |
| P00966 | ASSY_HUMAN | -1.5 | 2.1E-04 | 0.0028 |
| Q13404 | UB2V1_HUMAN | -1.5 | 3.5E-03 | 0.0163 |
| O15230 | LAMA5_HUMAN | -1.5 | 8.8E-03 | 0.0296 |
| Q8N335 | GPD1L_HUMAN | -1.4 | 7.8E-04 | 0.0055 |
| Q6PCB0 | VWA1_HUMAN | -1.4 | 5.9E-03 | 0.0230 |
| P43251 | BTD_HUMAN | -1.4 | 8.5E-06 | 0.0013 |
| P07942 | LAMB1_HUMAN | -1.4 | 2.8E-02 | 0.0756 |
| Q9H8H3 | MET7A_HUMAN | -1.4 | 1.2E-03 | 0.0079 |
| P51580 | TPMT_HUMAN | -1.4 | 8.6E-06 | 0.0011 |
| Q13057 | COASY_HUMAN | -1.4 | 2.7E-02 | 0.0740 |
| P15291 | B4GT1_HUMAN | -1.4 | 3.6E-02 | 0.0939 |
| P16422 | EPCAM_HUMAN | -1.4 | 4.4E-04 | 0.0039 |
| P11047 | LAMC1_HUMAN | -1.4 | 5.5E-03 | 0.0223 |
| P05026 | AT1B1_HUMAN | -1.4 | 2.5E-03 | 0.0128 |
| O00584 | RNT2_HUMAN | -1.4 | 2.5E-03 | 0.0128 |
| Q14165 | MLEC_HUMAN | -1.3 | 9.5E-06 | 0.0009 |
| Q02790 | FKBP4_HUMAN | -1.3 | 2.1E-04 | 0.0029 |
| Q02338 | BDH_HUMAN | -1.3 | 4.9E-02 | 0.1265 |
| P14625 | ENPL_HUMAN | -1.3 | 1.4E-02 | 0.0444 |
| P30086 | PEBP1_HUMAN | -1.3 | 7.3E-04 | 0.0053 |
| Q9Y4L1 | HYOU1_HUMAN | -1.3 | 8.4E-03 | 0.0287 |
| P11766 | ADHX_HUMAN | -1.3 | 1.6E-02 | 0.0491 |
| Q9NZJ7 | MTCH1_HUMAN | -1.2 | 1.2E-03 | 0.0078 |
| P55268 | LAMB2_HUMAN | -1.2 | 6.1E-03 | 0.0230 |
| P00352 | AL1A1_HUMAN | -1.2 | 3.2E-02 | 0.0832 |
| O14495 | LPP3_HUMAN | -1.2 | 3.1E-03 | 0.0148 |
| O00468 | AGRIN_HUMAN | -1.2 | 6.9E-03 | 0.0246 |
| P05091 | ALDH2_HUMAN | -1.2 | 8.6E-03 | 0.0290 |
| P08572 | CO4A2_HUMAN | -1.2 | 5.0E-02 | 0.1286 |
| P27797 | CALR_HUMAN | -1.2 | 5.5E-03 | 0.0225 |
| Q13425 | SNTB2_HUMAN | -1.1 | 4.5E-04 | 0.0040 |
| P23526 | SAHH_HUMAN | -1.1 | 3.3E-05 | 0.0011 |
| Q9GZM7 | TINAL_HUMAN | -1.1 | 3.6E-02 | 0.0945 |
| P11021 | GRP78_HUMAN | -1.1 | 2.1E-02 | 0.0618 |
| P35080 | PROF2_HUMAN | -1.1 | 2.2E-05 | 0.0010 |
| Q08380 | LG3BP_HUMAN | -1.1 | 7.9E-03 | 0.0270 |
| P21926 | CD9_HUMAN | -1.1 | 9.3E-04 | 0.0065 |
| P12830 | CADH1_HUMAN | -1.1 | 1.6E-03 | 0.0090 |
| Q15437 | SC23B_HUMAN | -1.1 | 1.3E-03 | 0.0079 |
| Q8TC12 | RDH11_HUMAN | -1.1 | 4.5E-03 | 0.0193 |
| Q06830 | PRDX1_HUMAN | -1.1 | 1.2E-02 | 0.0380 |
| P39060 | COIA1_HUMAN | -1.0 | 4.4E-03 | 0.0192 |
| Q9H0W9 | CK054_HUMAN | -1.0 | 3.5E-04 | 0.0033 |
| P05023 | AT1A1_HUMAN | -1.0 | 6.0E-03 | 0.0230 |
| Q13884 | SNTB1_HUMAN | -1.0 | 2.4E-02 | 0.0682 |
| Q9UMX0 | UBQL1_HUMAN | -1.0 | 1.4E-02 | 0.0449 |
| P16435 | NCPR_HUMAN | -1.0 | 4.7E-02 | 0.1209 |
| P23368 | MAOM_HUMAN | 1.0 | 1.9E-02 | 0.0566 |
| P60709 | ACTB_HUMAN | 1.0 | 2.5E-03 | 0.0127 |
| Q8IV08 | PLD3_HUMAN | 1.1 | 8.5E-03 | 0.0290 |
| Q13596 | SNX1_HUMAN | 1.1 | 3.9E-04 | 0.0034 |
| P01766 | HV305_HUMAN | 1.1 | 2.5E-02 | 0.0688 |
| Q9BTV4 | TMM43_HUMAN | 1.1 | 6.7E-03 | 0.0241 |
| O15143 | ARC1B_HUMAN | 1.1 | 1.8E-04 | 0.0024 |
| P06396 | GELS_HUMAN | 1.1 | 2.3E-04 | 0.0029 |
| Q9ULV4 | COR1C_HUMAN | 1.1 | 6.2E-03 | 0.0229 |
| Q99536 | VAT1_HUMAN | 1.1 | 3.0E-04 | 0.0033 |
| P37802 | TAGL2_HUMAN | 1.1 | 1.5E-03 | 0.0086 |
| P31949 | S10AB_HUMAN | 1.2 | 1.3E-03 | 0.0081 |
| P09382 | LEG1_HUMAN | 1.2 | 5.8E-03 | 0.0232 |
| P40121 | CAPG_HUMAN | 1.2 | 2.3E-02 | 0.0669 |
| P04433 | KV309_HUMAN | 1.2 | 2.2E-04 | 0.0027 |
| P25774 | CATS_HUMAN | 1.2 | 4.4E-03 | 0.0191 |
| P80723 | BASP1_HUMAN | 1.2 | 1.8E-03 | 0.0097 |
| P18135 | KV312_HUMAN | 1.3 | 3.6E-03 | 0.0167 |
| O60437 | PEPL_HUMAN | 1.3 | 7.7E-04 | 0.0056 |
| Q9Y6N5 | SQRD_HUMAN | 1.3 | 5.3E-03 | 0.0217 |
| P26038 | MOES_HUMAN | 1.3 | 2.3E-05 | 0.0009 |
| P01743 | HV102_HUMAN | 1.3 | 3.2E-03 | 0.0148 |
| Q96C19 | EFHD2_HUMAN | 1.3 | 7.5E-03 | 0.0262 |
| P53634 | CATC_HUMAN | 1.3 | 1.0E-02 | 0.0328 |
| O75223 | GGCT_HUMAN | 1.4 | 2.1E-03 | 0.0112 |
| Q9H299 | SH3L3_HUMAN | 1.4 | 3.6E-02 | 0.0941 |
| Q9H3G5 | CPVL_HUMAN | 1.4 | 2.4E-02 | 0.0673 |
| P00488 | F13A_HUMAN | 1.5 | 1.4E-02 | 0.0450 |
| P19971 | TYPH_HUMAN | 1.5 | 2.2E-02 | 0.0632 |
| P84095 | RHOG_HUMAN | 1.5 | 1.2E-02 | 0.0380 |
| P63313 | TYB10_HUMAN | 1.5 | 1.8E-07 | 0.0000 |
| Q14376 | GALE_HUMAN | 1.5 | 3.9E-03 | 0.0171 |
| P24821 | TENA_HUMAN | 1.5 | 1.2E-02 | 0.0372 |
| Q14764 | MVP_HUMAN | 1.6 | 6.1E-05 | 0.0009 |
| P52566 | GDIR2_HUMAN | 1.6 | 1.8E-03 | 0.0099 |
| Q9UH62 | ARMX3_HUMAN | 1.6 | 1.9E-04 | 0.0022 |
| Q13576 | IQGA2_HUMAN | 1.6 | 5.5E-05 | 0.0010 |
| P49961 | ENTP1_HUMAN | 1.6 | 2.6E-03 | 0.0130 |
| Q95IE3 | 2B1C_HUMAN | 1.7 | 3.5E-03 | 0.0163 |
| P36269 | GGT5_HUMAN | 1.7 | 2.1E-02 | 0.0607 |
| Q15121 | PEA15_HUMAN | 1.7 | 3.3E-04 | 0.0035 |
| P50479 | PDLI4_HUMAN | 1.8 | 8.9E-03 | 0.0296 |
| P06702 | S10A9_HUMAN | 1.8 | 2.0E-02 | 0.0594 |
| P07355 | ANXA2_HUMAN | 1.8 | 4.7E-03 | 0.0200 |
| P31947 | 1433S_HUMAN | 1.9 | 1.2E-03 | 0.0078 |
| Q9Y3Z3 | SAMH1_HUMAN | 1.9 | 4.7E-04 | 0.0041 |
| P42224 | STAT1_HUMAN | 1.9 | 5.5E-04 | 0.0044 |
| P04233 | HG2A_HUMAN | 1.9 | 5.5E-04 | 0.0046 |
| P42765 | THIM_HUMAN | 1.9 | 1.4E-02 | 0.0448 |
| P16144 | ITB4_HUMAN | 2.0 | 1.7E-03 | 0.0097 |
| P21980 | TGM2_HUMAN | 2.0 | 2.6E-03 | 0.0127 |
| Q86UX7 | URP2_HUMAN | 2.1 | 1.5E-03 | 0.0086 |
| P05109 | S10A8_HUMAN | 2.2 | 2.6E-03 | 0.0128 |
| Q14195 | DPYL3_HUMAN | 2.2 | 1.1E-03 | 0.0074 |
| P08727 | K1C19_HUMAN | 2.3 | 1.8E-02 | 0.0539 |
| Q96CX2 | KCD12_HUMAN | 2.4 | 1.2E-03 | 0.0078 |
| P01857 | IGHG1_HUMAN | 2.4 | 5.9E-05 | 0.0010 |
| P62328 | TYB4_HUMAN | 2.5 | 1.9E-05 | 0.0011 |
| P10316 | 1A69_HUMAN | 2.5 | 2.8E-02 | 0.0758 |
| P09758 | TACD2_HUMAN | 2.5 | 1.5E-02 | 0.0478 |
| P04083 | ANXA1_HUMAN | 2.5 | 8.4E-05 | 0.0009 |
| P31146 | COR1A_HUMAN | 2.6 | 6.3E-04 | 0.0044 |
| P17931 | LEG3_HUMAN | 2.6 | 9.0E-07 | 0.0000 |
| P05362 | ICAM1_HUMAN | 2.7 | 6.3E-05 | 0.0009 |
| O95571 | ETHE1_HUMAN | 2.7 | 1.9E-04 | 0.0023 |
| P09668 | CATH_HUMAN | 2.7 | 8.6E-03 | 0.0290 |
| Q8N6C5 | IGSF1_HUMAN | 2.8 | 2.5E-02 | 0.0711 |
| P13796 | PLSL_HUMAN | 2.9 | 1.0E-04 | 0.0011 |
| Q9UBG0 | MRC2_HUMAN | 3.1 | 1.9E-03 | 0.0102 |
| P15153 | RAC2_HUMAN | 3.4 | 1.8E-03 | 0.0098 |
| P02751 | FINC_HUMAN | 3.4 | 5.8E-04 | 0.0044 |
| Q8WU39 | MZB1_HUMAN | 3.4 | 1.1E-03 | 0.0074 |

**Supplementary Table S6.** Common proteins differentially expressed between FTC/N and PTC/N as determined by SWATH

| Uniprot | Protein | **N-FTC log2(fc)** | **N-PTC log2(fc)** |
| --- | --- | --- | --- |
| O14498 | ISLR_HUMAN | -3.4 | -3.5 |
| P01266 | THYG_HUMAN | -3.2 | -6.2 |
| P68871 | HBB_HUMAN | -2.9 | -2 |
| P02743 | SAMP_HUMAN | -2.9 | -3.3 |
| P02042 | HBD_HUMAN | -2.9 | -2.2 |
| P22748 | CAH4_HUMAN | -2.8 | -4 |
| P69905 | HBA_HUMAN | -2.8 | -2.1 |
| Q8N5Z5 | KCD17_HUMAN | -2.8 | -6.6 |
| P00915 | CAH1_HUMAN | -2.6 | -1.5 |
| Q07654 | TFF3_HUMAN | -2.6 | -7.1 |
| P02730 | B3AT_HUMAN | -2.5 | -2 |
| P36269 | GGT5_HUMAN | -2.5 | -1 |
| P08294 | SODE_HUMAN | -2.4 | -4.4 |
| P11166 | GTR1_HUMAN | -2.1 | -1.5 |
| Q16853 | AOC3_HUMAN | -2.1 | -1.8 |
| P22105 | TENX_HUMAN | -2.1 | -1 |
| P11277 | SPTB1_HUMAN | -1.9 | -1.9 |
| P02679 | FIBG_HUMAN | -1.8 | -1.3 |
| P01023 | A2MG_HUMAN | -1.7 | -1.2 |
| P17661 | DESM_HUMAN | -1.6 | -2.3 |
| Q86XX4 | FRAS1_HUMAN | -1.6 | -2.9 |
| P00918 | CAH2_HUMAN | -1.5 | -1.3 |
| Q86UN3 | R4RL2_HUMAN | -1.5 | -3.3 |
| Q9NZN4 | EHD2_HUMAN | -1.5 | -1.3 |
| Q9H223 | EHD4_HUMAN | -1.4 | -1.6 |
| P07738 | PMGE_HUMAN | -1.4 | -1.3 |
| Q9BX97 | PLVAP_HUMAN | -1.3 | -2 |
| Q6NZI2 | PTRF_HUMAN | -1.3 | -1.4 |
| P30043 | BLVRB_HUMAN | -1.3 | -1.3 |
| P43251 | BTD_HUMAN | -1.3 | -2.7 |
| Q9Y646 | CBPQ_HUMAN | -1.3 | -2.9 |
| P02511 | CRYAB_HUMAN | -1.2 | -2.9 |
| P32119 | PRDX2_HUMAN | -1.2 | -1.2 |
| Q13884 | SNTB1_HUMAN | -1.0 | -2 |
| P10768 | ESTD_HUMAN | -1.0 | -1.3 |
| P50454 | SERPH_HUMAN | 1.2 | 1.4 |
| P61981 | 1433G_HUMAN | 1.4 | 1.6 |
| P24821 | TENA_HUMAN | 1.5 | 2.5 |
| O95994 | AGR2_HUMAN | 2.0 | 3.4 |

**Supplementary Table S7.** iTRAQ confirmation of differential protein expression in PTC/FTC

| **Uniprot** | **Protein** | **iTRAQ** | **iTRAQ**  **ratio** | **SWATH**  **log2(fc)** |
| --- | --- | --- | --- | --- |
|  |  | **p-value** |  |  |
| P02751 | FINC_HUMAN | 1.70E-03 | 4.89 | 3.4 |
| P15153 | RAC2_HUMAN | 2.40E-02 | 2.99 | 3.4 |
| P13796 | PLSL_HUMAN | 4.20E-03 | 2.47 | 2.9 |
| Q8N6C5 | IGSF1_HUMAN | 4.10E-02 | 2.01 | 2.8 |
| O95571 | ETHE1_HUMAN | 1.10E-05 | 3.12 | 2.7 |
| P05362 | ICAM1_HUMAN | 8.70E-04 | 1.69 | 2.7 |
| P17931 | LEG3_HUMAN | 1.50E-06 | 3.45 | 2.6 |
| P31146 | COR1A_HUMAN | 4.80E-03 | 2.45 | 2.6 |
| P04083 | ANXA1_HUMAN | 2.40E-04 | 5.37 | 2.5 |
| P09758 | TACD2_HUMAN | 8.80E-03 | 1.96 | 2.5 |
| P62328 | TYB4_HUMAN | 7.50E-04 | 3.29 | 2.5 |
| Q96CX2 | KCD12_HUMAN | 3.30E-02 | 1.54 | 2.4 |
| P08727 | K1C19_HUMAN | 1.70E-02 | 2.8 | 2.3 |
| Q14195 | DPYL3_HUMAN | 3.80E-03 | 2.11 | 2.2 |
| P05109 | S10A8_HUMAN | 1.10E-02 | 2.05 | 2.2 |
| P21980 | TGM2_HUMAN | 3.80E-02 | 1.93 | 2 |
| P04233 | HG2A_HUMAN | 3.50E-03 | 1.65 | 1.9 |
| P42224 | STAT1_HUMAN | 1.40E-02 | 1.66 | 1.9 |
| Q9Y3Z3 | SAMH1_HUMAN | 3.40E-02 | 1.39 | 1.9 |
| P07355 | ANXA2_HUMAN | 3.90E-03 | 2.46 | 1.8 |
| P06702 | S10A9_HUMAN | 1.30E-02 | 2.29 | 1.8 |
| P36269 | GGT5_HUMAN | 4.70E-02 | 1.55 | 1.7 |
| P52566 | GDIR2_HUMAN | 3.00E-02 | 1.65 | 1.6 |
| Q14764 | MVP_HUMAN | 1.50E-05 | 1.79 | 1.6 |
| P63313 | TYB10_HUMAN | 1.90E-06 | 2.32 | 1.5 |
| P84095 | RHOG_HUMAN | 2.60E-02 | 1.74 | 1.5 |
| P19971 | TYPH_HUMAN | 5.60E-03 | 1.82 | 1.5 |
| P00488 | F13A_HUMAN | 4.20E-02 | 1.51 | 1.5 |
| Q9H299 | SH3L3_HUMAN | 5.60E-04 | 2.23 | 1.4 |
| O75223 | GGCT_HUMAN | 1.70E-03 | 2.58 | 1.4 |
| P53634 | CATC_HUMAN | 8.30E-03 | 1.59 | 1.3 |
| Q96C19 | EFHD2_HUMAN | 2.90E-03 | 1.65 | 1.3 |
| P26038 | MOES_HUMAN | 1.00E-05 | 1.94 | 1.3 |
| Q9Y6N5 | SQRD_HUMAN | 3.00E-02 | 1.44 | 1.3 |
| O60437 | PEPL_HUMAN | 4.10E-04 | 1.57 | 1.3 |
| P80723 | BASP1_HUMAN | 6.90E-03 | 1.72 | 1.2 |
| P04433 | KV309_HUMAN | 9.00E-04 | 1.96 | 1.2 |
| P40121 | CAPG_HUMAN | 4.50E-02 | 1.69 | 1.2 |
| P09382 | LEG1_HUMAN | 3.20E-02 | 1.85 | 1.2 |
| P31949 | S10AB_HUMAN | 5.30E-04 | 2.17 | 1.2 |
| P37802 | TAGL2_HUMAN | 5.30E-03 | 1.63 | 1.1 |
| Q99536 | VAT1_HUMAN | 2.20E-03 | 1.56 | 1.1 |
| P06396 | GELS_HUMAN | 1.00E-03 | 1.75 | 1.1 |
| O15143 | ARC1B_HUMAN | 9.20E-05 | 1.68 | 1.1 |
| Q9BTV4 | TMM43_HUMAN | 5.70E-04 | 1.53 | 1.1 |
| Q13596 | SNX1_HUMAN | 5.90E-03 | 1.5 | 1.1 |
| P05023 | AT1A1_HUMAN | 3.30E-02 | -1.52 | -1 |
| P39060 | COIA1_HUMAN | 1.70E-02 | -1.58 | -1 |
| Q06830 | PRDX1_HUMAN | 2.10E-02 | -1.7 | -1.1 |
| P12830 | CADH1_HUMAN | 1.80E-02 | -1.38 | -1.1 |
| P21926 | CD9_HUMAN | 4.80E-03 | -2.25 | -1.1 |
| Q08380 | LG3BP_HUMAN | 2.40E-02 | -1.95 | -1.1 |
| P11021 | GRP78_HUMAN | 3.20E-02 | -1.85 | -1.1 |
| P23526 | SAHH_HUMAN | 2.00E-03 | -1.5 | -1.1 |
| P27797 | CALR_HUMAN | 7.30E-03 | -2.11 | -1.2 |
| P05091 | ALDH2_HUMAN | 7.50E-03 | -1.68 | -1.2 |
| O00468 | AGRIN_HUMAN | 3.00E-02 | -1.54 | -1.2 |
| O14495 | LPP3_HUMAN | 2.10E-04 | -2.47 | -1.2 |
| P00352 | AL1A1_HUMAN | 1.70E-02 | -1.84 | -1.2 |
| P55268 | LAMB2_HUMAN | 1.00E-02 | -1.45 | -1.2 |
| P11766 | ADHX_HUMAN | 2.00E-03 | -1.41 | -1.3 |
| Q9Y4L1 | HYOU1_HUMAN | 2.10E-02 | -1.71 | -1.3 |
| P30086 | PEBP1_HUMAN | 4.20E-04 | -1.88 | -1.3 |
| P14625 | ENPL_HUMAN | 2.20E-02 | -2.13 | -1.3 |
| Q02790 | FKBP4_HUMAN | 5.60E-04 | -1.37 | -1.3 |
| Q14165 | MLEC_HUMAN | 1.80E-02 | -1.51 | -1.3 |
| O00584 | RNT2_HUMAN | 2.60E-03 | -2.01 | -1.4 |
| P05026 | AT1B1_HUMAN | 1.40E-02 | -1.92 | -1.4 |
| P11047 | LAMC1_HUMAN | 1.10E-02 | -1.7 | -1.4 |
| P16422 | EPCAM_HUMAN | 4.40E-03 | -1.48 | -1.4 |
| Q9H8H3 | MET7A_HUMAN | 5.50E-04 | -1.99 | -1.4 |
| Q6PCB0 | VWA1_HUMAN | 3.80E-02 | -1.43 | -1.4 |
| O15230 | LAMA5_HUMAN | 2.90E-02 | -1.81 | -1.5 |
| P00966 | ASSY_HUMAN | 1.00E-02 | -1.33 | -1.5 |
| P30038 | AL4A1_HUMAN | 4.90E-02 | -1.22 | -1.6 |
| Q14112 | NID2_HUMAN | 4.40E-02 | -1.61 | -1.6 |
| Q13228 | SBP1_HUMAN | 4.60E-03 | -2.17 | -1.7 |
| Q9Y646 | CBPQ_HUMAN | 2.60E-02 | -2.15 | -1.7 |
| P23434 | GCSH_HUMAN | 8.50E-04 | -2.12 | -1.7 |
| Q96CM8 | ACSF2_HUMAN | 7.80E-03 | -1.58 | -1.7 |
| P02511 | CRYAB_HUMAN | 1.00E-02 | -1.7 | -1.7 |
| Q16363 | LAMA4_HUMAN | 9.70E-03 | -1.51 | -1.8 |
| Q6UXI9 | NPNT_HUMAN | 1.80E-03 | -2.47 | -1.9 |
| P50895 | BCAM_HUMAN | 1.90E-04 | -2.01 | -1.9 |
| Q13642 | FHL1_HUMAN | 3.00E-06 | -1.98 | -2 |
| P08294 | SODE_HUMAN | 2.70E-02 | -2.1 | -2 |
| Q6PHW0 | IYD1_HUMAN | 3.40E-05 | -2.78 | -2 |
| O94875 | SRBS2_HUMAN | 1.70E-03 | -1.69 | -2.2 |
| Q5SZK8 | FREM2_HUMAN | 5.70E-03 | -2.7 | -2.2 |
| O00339 | MATN2_HUMAN | 2.20E-02 | -2.18 | -2.2 |
| Q00796 | DHSO_HUMAN | 5.50E-03 | -3.59 | -2.4 |
| P22352 | GPX3_HUMAN | 3.20E-02 | -3.36 | -2.4 |
| P21266 | GSTM3_HUMAN | 1.10E-02 | -2.4 | -2.6 |
| P12277 | KCRB_HUMAN | 5.40E-03 | -4.33 | -3.5 |
| P07202 | PERT_HUMAN | 7.10E-03 | -3.31 | -4.1 |

**Supplementary Table S8. Peptide sequences and transitions employed for MRM-HR quantitation of selected proteins in histologically normal, FA, FTC and PTC samples**

| Protein | Peptide | Precursor m/z | Charge | Collision  energy | Product  m/z | Charge | Fragment  Ion |
| --- | --- | --- | --- | --- | --- | --- | --- |
| RAB7A_HUMAN | VIILGDSGVGK | 529.3162 | 2 | 18.8 | 845.4727 | 1 | y9 |
| RAB7A_HUMAN | VIILGDSGVGK | 529.3162 | 2 | 18.8 | 732.3886 | 1 | y8 |
| RAB7A_HUMAN | VIILGDSGVGK | 529.3162 | 2 | 18.8 | 619.3046 | 1 | y7 |
| RAB7A_HUMAN | FQSLGVAFYR | 594.3140 | 2 | 20.7 | 912.4938 | 1 | y8 |
| RAB7A_HUMAN | FQSLGVAFYR | 594.3140 | 2 | 20.7 | 825.4618 | 1 | y7 |
| RAB7A_HUMAN | FQSLGVAFYR | 594.3140 | 2 | 20.7 | 712.3777 | 1 | y6 |
| PPIA_HUMAN | VSFELFADK | 528.2740 | 2 | 18.8 | 956.4724 | 1 | y8 |
| PPIA_HUMAN | VSFELFADK | 528.2740 | 2 | 18.8 | 869.4403 | 1 | y7 |
| PPIA_HUMAN | VSFELFADK | 528.2740 | 2 | 18.8 | 722.3719 | 1 | y6 |
| PPIA_HUMAN | FEDENFILK | 577.7900 | 2 | 20.2 | 1007.5044 | 1 | y8 |
| PPIA_HUMAN | FEDENFILK | 577.7900 | 2 | 20.2 | 878.4618 | 1 | y7 |
| PPIA_HUMAN | FEDENFILK | 577.7900 | 2 | 20.2 | 763.4349 | 1 | y6 |
| ARC1B_HUMAN | ASSEGGTAAGAGLDSLHK | 543.6007 | 3 | 22.9 | 968.5160 | 1 | y10 |
| ARC1B_HUMAN | ASSEGGTAAGAGLDSLHK | 543.6007 | 3 | 22.9 | 897.4789 | 1 | y9 |
| ARC1B_HUMAN | ASSEGGTAAGAGLDSLHK | 543.6007 | 3 | 22.9 | 769.4203 | 1 | y7 |
| ARC1B_HUMAN | NSVSQISVLSGGK | 638.3488 | 2 | 22.1 | 975.5469 | 1 | y10 |
| ARC1B_HUMAN | NSVSQISVLSGGK | 638.3488 | 2 | 22.1 | 760.4563 | 1 | y8 |
| ARC1B_HUMAN | NSVSQISVLSGGK | 638.3488 | 2 | 22.1 | 647.3723 | 1 | y7 |
| CADH1_HUMAN | TAYFSLDTR | 537.2667 | 2 | 19 | 901.4414 | 1 | y7 |
| CADH1_HUMAN | TAYFSLDTR | 537.2667 | 2 | 19 | 738.3781 | 1 | y6 |
| CADH1_HUMAN | TAYFSLDTR | 537.2667 | 2 | 19 | 591.3097 | 1 | y5 |
| CADH1_HUMAN | TIFFC[+57]ER | 486.7340 | 2 | 17.5 | 758.3290 | 1 | y5 |
| CADH1_HUMAN | TIFFC[+57]ER | 486.7340 | 2 | 17.5 | 611.2606 | 1 | y4 |
| CADH1_HUMAN | TIFFC[+57]ER | 486.7340 | 2 | 17.5 | 464.1922 | 1 | y3 |
| ARPC4_HUMAN | ELLLQPVTISR | 634.8823 | 2 | 22 | 913.5465 | 1 | y8 |
| ARPC4_HUMAN | ELLLQPVTISR | 634.8823 | 2 | 22 | 800.4625 | 1 | y7 |
| ARPC4_HUMAN | ELLLQPVTISR | 634.8823 | 2 | 22 | 672.4039 | 1 | y6 |
| ARPC4_HUMAN | VLIEGSINSVR | 593.8431 | 2 | 20.7 | 974.5265 | 1 | y9 |
| ARPC4_HUMAN | VLIEGSINSVR | 593.8431 | 2 | 20.7 | 861.4425 | 1 | y8 |
| ARPC4_HUMAN | VLIEGSINSVR | 593.8431 | 2 | 20.7 | 732.3999 | 1 | y7 |
| TFF3_HUMAN | EEYVGLSANQC[+57]AVPAKDR | 669.6582 | 3 | 27.7 | 916.4669 | 1 | y8 |
| TFF3_HUMAN | EEYVGLSANQC[+57]AVPAKDR | 669.6582 | 3 | 27.7 | 756.4363 | 1 | y7 |
| TFF3_HUMAN | EEYVGLSANQC[+57]AVPAKDR | 669.6582 | 3 | 27.7 | 586.3307 | 1 | y5 |
| BTD_HUMAN | LSSGLVTAALYGR | 654.3695 | 2 | 22.5 | 1107.6157 | 1 | y11 |
| BTD_HUMAN | LSSGLVTAALYGR | 654.3695 | 2 | 22.5 | 850.4781 | 1 | y8 |
| BTD_HUMAN | LSSGLVTAALYGR | 654.3695 | 2 | 22.5 | 751.4097 | 1 | y7 |
| SODE_HUMAN | AVVVHAGEDDLGR | 446.5651 | 3 | 19.3 | 969.4384 | 1 | y9 |
| SODE_HUMAN | AVVVHAGEDDLGR | 446.5651 | 3 | 19.3 | 832.3795 | 1 | y8 |
| SODE_HUMAN | AVVVHAGEDDLGR | 446.5651 | 3 | 19.3 | 761.3424 | 1 | y7 |
| AGR2_HUMAN | IMFVDPSLTVR | 639.3497 | 2 | 22.1 | 1033.5677 | 1 | y9 |
| AGR2_HUMAN | IMFVDPSLTVR | 639.3497 | 2 | 22.1 | 787.4308 | 1 | y7 |
| AGR2_HUMAN | IMFVDPSLTVR | 639.3497 | 2 | 22.1 | 672.4039 | 1 | y6 |
| AGR2_HUMAN | LYAYEPADTALLLDNMKK | 690.3590 | 3 | 28.5 | 1429.7719 | 1 | y13 |
| AGR2_HUMAN | LYAYEPADTALLLDNMKK | 690.3590 | 3 | 28.5 | 861.4863 | 1 | y7 |
| AGR2_HUMAN | LYAYEPADTALLLDNMKK | 690.3590 | 3 | 28.5 | 748.4022 | 1 | y6 |
| PERT_HUMAN | LPEPTSGVIAR | 570.3246 | 2 | 20 | 929.5051 | 1 | y9 |
| PERT_HUMAN | LPEPTSGVIAR | 570.3246 | 2 | 20 | 800.4625 | 1 | y8 |
| PERT_HUMAN | LPEPTSGVIAR | 570.3246 | 2 | 20 | 703.4097 | 1 | y7 |
| BGH3_HUMAN | LTLLAPLNSVFK | 658.4028 | 2 | 22.7 | 988.5826 | 1 | y9 |
| BGH3_HUMAN | LTLLAPLNSVFK | 658.4028 | 2 | 22.7 | 875.4985 | 1 | y8 |
| BGH3_HUMAN | LTLLAPLNSVFK | 658.4028 | 2 | 22.7 | 804.4614 | 1 | y7 |
| BGH3_HUMAN | EGVYTVFAPTNEAFR | 850.9176 | 2 | 28.4 | 1052.5160 | 1 | y9 |
| BGH3_HUMAN | EGVYTVFAPTNEAFR | 850.9176 | 2 | 28.4 | 905.4476 | 1 | y8 |
| BGH3_HUMAN | EGVYTVFAPTNEAFR | 850.9176 | 2 | 28.4 | 834.4104 | 1 | y7 |
| TENA_HUMAN | VATYLPAPEGLK | 629.8557 | 2 | 21.8 | 824.4876 | 1 | y8 |
| TENA_HUMAN | VATYLPAPEGLK | 629.8557 | 2 | 21.8 | 711.4036 | 1 | y7 |
| TENA_HUMAN | VATYLPAPEGLK | 629.8557 | 2 | 21.8 | 543.3137 | 1 | y5 |
| SERPH_HUMAN | LYGPSSVSFADDFVR | 830.4043 | 2 | 27.8 | 1383.6539 | 1 | y13 |
| SERPH_HUMAN | LYGPSSVSFADDFVR | 830.4043 | 2 | 27.8 | 1055.5156 | 1 | y9 |
| SERPH_HUMAN | LYGPSSVSFADDFVR | 830.4043 | 2 | 27.8 | 956.4472 | 1 | y8 |
| SERPH_HUMAN | DTQSGSLLFIGR | 647.3435 | 2 | 22.3 | 949.5465 | 1 | y9 |
| SERPH_HUMAN | DTQSGSLLFIGR | 647.3435 | 2 | 22.3 | 862.5145 | 1 | y8 |
| SERPH_HUMAN | DTQSGSLLFIGR | 647.3435 | 2 | 22.3 | 605.3770 | 1 | y5 |
| PGS2_HUMAN | VVQC[+57]SDLGLDKVPK | 519.9484 | 3 | 22 | 1071.6045 | 1 | y10 |
| PGS2_HUMAN | VVQC[+57]SDLGLDKVPK | 519.9484 | 3 | 22 | 869.5455 | 1 | y8 |
| PGS2_HUMAN | VVQC[+57]SDLGLDKVPK | 519.9484 | 3 | 22 | 756.4614 | 1 | y7 |
| PGS2_HUMAN | VSPGAFTPLVK | 558.3266 | 2 | 19.7 | 929.5455 | 1 | y9 |
| PGS2_HUMAN | VSPGAFTPLVK | 558.3266 | 2 | 19.7 | 775.4713 | 1 | y7 |
| PGS2_HUMAN | VSPGAFTPLVK | 558.3266 | 2 | 19.7 | 465.2764 | 2 | y9 |
